# Transplantation of rat embryonic stem cell-derived retinal progenitor cells preserves the retinal structure and function in rat retinal degeneration

**DOI:** 10.1186/s13287-015-0207-x

**Published:** 2015-11-09

**Authors:** Zepeng Qu, Yuan Guan, Lu Cui, Jian Song, Junjie Gu, Hanzhi Zhao, Lei Xu, Lixia Lu, Ying Jin, Guo-Tong Xu

**Affiliations:** Laboratory of Molecular Developmental Biology, Shanghai Jiao Tong University School of Medicine, Room 208, Building 5, 280 South Chongqing Road, Shanghai, 200025 China; Department of Ophthalmology of Shanghai Tenth People’s Hospital, and Laboratory of Clinical Visual Science of Tongji Eye Institute, Tongji University School of Medicine, 1239 Siping Road, Medical Building, Room 521, Shanghai, 200092 China; Key Laboratory of Stem Cell Biology, Institute of Health Sciences, Shanghai Institute for Biological Sciences, Chinese Academy of Sciences/Shanghai Jiao Tong University School of Medicine, Shanghai, 200031 China; ShanghaiTech University School of Life Science and Technology, Shanghai, 201210 China; Department of Regenerative Medicine, Stem Cell Research Center, and Institute for Nutritional Sciences, Tongji University School of Medicine, Shanghai, 200092 China

**Keywords:** Rat embryonic stem cell, Degenerative retinal diseases, Retinal progenitor cell (RPC), Transplantation, Royal College of Surgeons (RCS) rat

## Abstract

**Introduction:**

Degenerative retinal diseases like age-related macular degeneration (AMD) are the leading cause of blindness. Cell transplantation showed promising therapeutic effect for such diseases, and embryonic stem cell (ESC) is one of the sources of such donor cells. Here, we aimed to generate retinal progenitor cells (RPCs) from rat ESCs (rESCs) and to test their therapeutic effects in rat model.

**Methods:**

The rESCs (DA8-16) were cultured in N2B27 medium with 2i, and differentiated to two types of RPCs following the SFEBq method with modifications. For rESC-RPC1, the cells were switched to adherent culture at D10, while for rESC-RPC2, the suspension culture was maintained to D14. Both RPCs were harvested at D16. Primary RPCs were obtained from P1 SD rats, and some of them were labeled with EGFP by infection with lentivirus. To generate Rax::EGFP knock-in rESC lines, TALENs were engineered to facilitate homologous recombination in rESCs, which were cotransfected with the targeting vector and TALEN vectors. The differentiated cells were analyzed with live image, immunofluorescence staining, flow cytometric analysis, gene expression microarray, etc. RCS rats were used to mimic the degeneration of retina and test the therapeutic effects of subretinally transplanted donor cells. The structure and function of retina were examined.

**Results:**

We established two protocols through which two types of rESC-derived RPCs were obtained and both contained committed retina lineage cells and some neural progenitor cells (NPCs). These rESC-derived RPCs survived in the host retinas of RCS rats and protected the retinal structure and function in early stage following the transplantation. However, the glia enriched rESC-RPC1 obtained through early and longer adherent culture only increased the b-wave amplitude at 4 weeks, while the longer suspension culture gave rise to evidently neuronal differentiation in rESC-RPC2 which significantly improved the visual function of RCS rats.

**Conclusions:**

We have successfully differentiated rESCs to glia enriched RPCs and retinal neuron enriched RPCs *in vitro*. The retinal neuron enriched rESC-RPC2 protected the structure and function of retina in rats with genetic retinal degeneration and could be a candidate cell source for treating some degenerative retinal diseases in human trials.

**Electronic supplementary material:**

The online version of this article (doi:10.1186/s13287-015-0207-x) contains supplementary material, which is available to authorized users.

## Introduction

Degenerative retinal diseases, such as age-related macular degeneration (AMD) and retinitis pigmentosa (RP), are the leading causes of blindness in the developed world, and their common pathology includes the death of photoreceptors, which results in permanent loss of vision [1–3]. Although recent anti-vascular endothelial growth factor (VEGF) treatments showed an intervention effect in wet AMD [4, 5], there is basically no effective treatment to restore visual function for most of the patients with such diseases [6]. Now, cell-based therapy is considered to be a potential therapeutic approach [7–10]. Several types of desired donor cells, such as retinal pigment epithelium (RPE) cells, photoreceptors and retinal progenitor cells (RPCs), differentiated from different types of stem cells have been studied [11–13]. Transplantation of RPCs collected from newborn mouse retinas could effectively integrate into recipient retinas and restore photosensitivity in the mouse model of retinal degeneration [14, 15]. However, in clinic, the availability of primary RPCs (P-RPCs) from newborn individuals is almost impossible and its application would be restricted. Instead, differentiation of pluripotent or multipotent stem cells into RPCs or other retinal cells becomes a reasonable replacement of primary cells.

Embryonic stem cells (ESCs) are regarded as an attractive source of donor cells due to their pluripotency and unlimited expansion capacity *in vitro*, and have been widely researched for retinal lineage differentiation in mice and humans [16–21]. Transplantation of these ESC-derived cells in animal models with degenerative retinal disease has been proven a realistic approach to rescue visual function [7, 15, 22, 23]. In particular, human ESC-derived RPE cells were recently tested to treat patients suffering from degenerative retinal diseases and the visual acuities were improved in 10 out of 18 transplanted patients [7]. The trial for RPE cells derived from patient somatic cells with induced pluripotent stem (iPS) cell technology was also started in 2014 [24]. While these pluripotent stem cell-derived RPE cells bring hope to such patients, they also face some challenges. Transplantation of primary adult RPE cell sheets did not improve the vision in patients [25], challenging the therapeutic application of stem cell derived RPE cells. Furthermore, degenerative retinal diseases share the common pathology that is the death of photoreceptors. These photoreceptors are terminally differentiated and unrenewable neurons which are the key unit for vision [26]. Logically, RPE cell transplantation might not be sufficient to rescue vision in the cases in which most photoreceptors died [7, 27, 28], as occurs in late stage AMD. Therefore, replacement therapy with pluripotent stem cell (such as ESC)-derived retinal neural lineage cells including RPCs is regarded as a promising strategy for treating such diseases. But the ESC derivatives developed tumors despite predifferentiating or presorting the cells [15, 29, 30]. Only photoreceptor precursors repaired retinal defects efficiently without tumor formation [15, 31], indicating that the developmental stage of donor cells determined the cell fate following transplantation. In short, many important questions regarding stem cell therapy, such as how to improve its efficacy and assure its safety, deserve further investigation.

It is well known that the rat is a valuable experimental tool for modeling human diseases due to its relevance to humans [32, 33]. Moreover, as compared with the mouse eye, the larger eyeballs of rats were more convenient to use; for example, it makes our in vivo retinal observation easier and clearer, it makes the subretinal cell delivery more accurate, and we can inject more donor cells into the larger subretinal space to assure effective transplantation. Even though the murine and human ESC lines were established in 1981 and 1998 [34, 35], rat ESCs (rESCs) were not established until 2008 [36, 37], when the successful derivation of authentic rESC lines using a combination of serum-free culture and specific signal inhibitors was reported [32, 36, 37]. Until now, however, only a few other rESCs related studies have been reported [38–42]. The reports on differentiations of rESCs are even fewer and in those studies rESCs were differentiated into functional cardiomyocytes, tripotent neural progenitor cells and granulosa-like cells [38, 40, 42]. Although mouse ESCs can be used for study, it was reported that epiblasts of rat and mouse are distinct in intrinsic differentiation capacity [43]. It remains unclear whether rESCs could be efficiently differentiated into retinal cell lineages. Particularly, to date, no study on transplantation of rESC-derived cells in a disease animal model has been reported.

Rats have been used as models for many human diseases, such as neural disorders, diabetes, hypertension and heart failure, for scientific studies and drug discovery [44–47]. In this study, we investigated whether transplantation of rESC-derived RPCs (rESC-RPCs) could preserve visional function as well as retinal structure in the Royal College of Surgeons (RCS) rat, a well-established retinal degeneration model [48–50]. The RCS rat carries a *Mertk* gene mutation in the RPE cells [51] that fail to phagocytose and shed the outer segment of photoreceptors, causing the accumulation of outer segment debris and, subsequently, degeneration and loss of photoreceptors. As the model displays defects comparable to those of patients suffering from degenerative retinal diseases, it has served as a preclinical model for RP and AMD [52–54].

In this study, we differentiated rESCs into RPCs and transplanted these rESC-RPCs into the eye of RCS rats. The transplanted rESC-RPCs could survive in the host retina and protect the retinal structure. Moreover, the grafted cells integrate into the retina of rats and preserve the retinal function in the early stage after transplantation. Therefore, the study develops an approach for rESCs to differentiate into RPCs in vitro and provides the first example for the transplantation of rESC-RPCs in a disease model with positive intervention effects.

## Methods

### Rat embryonic stem cell culture and retinal progenitor cell differentiation

The rESC line DA8-16, a generous gift from Lei Xiao and Chun Cui (Zhejiang University School of Medicine), was cultured in N2B27 medium supplemented with 2i (MEK inhibitor: PD0325901, 0.4 μM, Stemgent, Cambridge, MA, USA; GSK3 inhibitor: CHIR99021, 3 μM, Stemgent) on gamma radiation-inactivated mouse embryonic fibroblast (MEF) feeder layers as described previously [38]. The medium was changed daily and rESCs were passaged every four to six days by dissociation with TrypLE Express (Gibco, Grand Island, NY, USA) into single cells and transferred onto inactivated MEF.

**Fig. 1:**
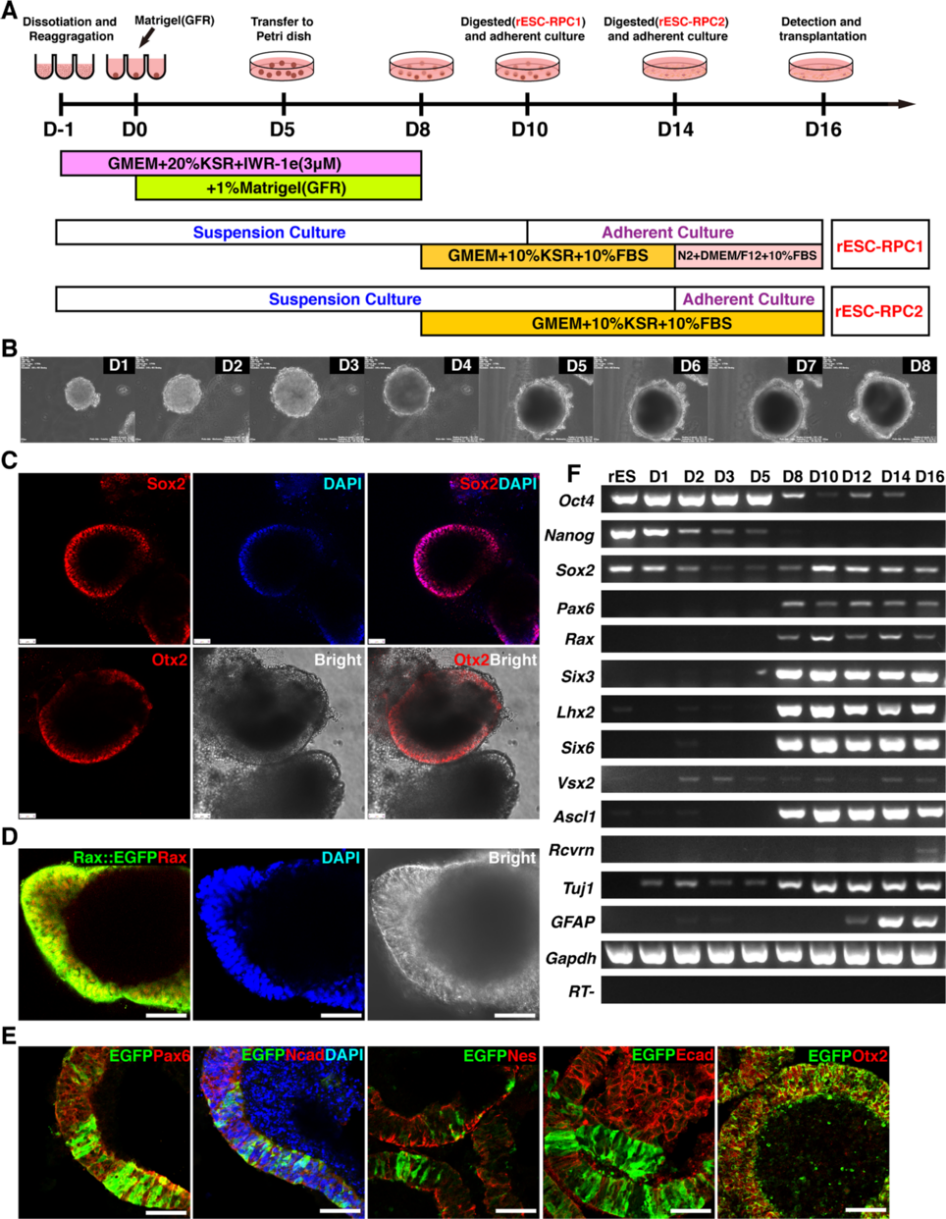
*In vitro* differentiation of rat ESCs into RPCs. **a** Schematic diagram illustrating the strategy for differentiating rESCs into RPCs in this study. **b** Images of morphological changes of differentiating rESCs from day 1 to the formation of neuroectoderm-like structure at day 8. **c** Whole mount immunofluorescence staining, using antibodies against Sox2 and Otx2 (*red*), and bright images of rESC-derived neuroectoderm-like structure at day 8. DAPI (*blue*) was used to highlight the nuclei. Scale bar: 50 μm. **d** Whole mount immunofluorescence staining using antibody against Rax (*red*) and bright images of Rax::EGFP rESC-derived neuroectoderm-like structure at day 8. DAPI (*blue*) was used to highlight the nuclei. Scale bar: 50 μm. **e** Immunofluorescence images of cryosection of the neuroectoderm-like structure derived from Rax::EGFP rESCs at day 8. Antibody against EGFP (*green*) and antibodies against Pax6, N-cadherin/Ncad, Nestin/Nes, E-cadherin/Ecad and Otx2 (*red*) were used. DAPI (*blue*) was used to highlight the nuclei. Scale bar: 50 μm. **f** A representative result of RT-PCR analyses for marker expression during the differentiation process (rESC-RPC2). r*ESCs* rat embryonic stem cells, *RPCs* retinal progenitor cells, *DAPI* 4′,6-diamidino-2-phenylindole, *EGFP* enhanced green fluorescent protein, *RT-PCR* reverse transcription polymerase chain reaction

For RPC differentiation, rESCs discarded feeder underwent differentiation following the quickly-aggregated serum-free embryonic body (SFEBq) method [17] with modifications. In detail, rESCs were dissociated into single cells in TrypLE Express containing DNase I (0.05 mg/ml, Sigma-Aldrich, Saint Louis, MO, USA), and were quickly reaggregated in neuroectoderm differentiation medium (5,000 cells/100 μl/well) using an ultra-low-attachment 96-well plate with U-bottom wells (Corning, Corning, NY, USA). The neuroectoderm differentiation medium was GMEM (Gibco) supplemented with 20 % Knockout Serum Replacement (KSR, Gibco), 0.1 mM nonessential amino acids (Gibco), 1 mM sodium pyruvate (Gibco), 0.1 mM 2-mercaptoethanol (Gibco), 3 μM wnt inhibitor IWR-1e (Merck, Darmstadt, Germany), 100 U/ml penicillin and 100 mg/ml streptomycin (Gibco). In the second day of cell aggregate formation, Matrigel (growth-factor-reduced; BD Biosciences, Bedford, MA, USA) was added to the medium (final 1 % v/v) and the day was defined as day 0 (D0). At D5, SFEBs were transferred from a 96-well plate to a low adherent Petri dish (BD Biosciences or Qingdao Alpha, Qingdao, Shandong, China) and the medium was changed to fresh neuroectoderm differentiation medium containing 1 % Matrigel (96 SFEBs per 10-cm dish). At D8, Matrigel was withdrawn from the culture and the medium was changed to retinal differentiation medium containing GMEM supplemented with 10 % KSR, 10 % FBS (Hyclone, Logan, UT, USA), 0.1 mM nonessential amino acids, 1 mM sodium pyruvate, 0.1 mM 2-mercaptoethanol, 100 U/ml penicillin and 100 mg/ml streptomycin. Two days later (D10), the SFEBs were digested and replated onto poly-D-lysine (PDL) (Millipore, Billerica, MA, USA) and Matrigel (BD Biosciences)-coated plates for further adherent culture (early adherent culture method). Retinal differentiation medium, containing DMEM/F12 (Gibco) medium with 1 % N2 supplement (Gibco), 10 % FBS, 0.1 mM nonessential amino acids, 1 mM sodium pyruvate, 100 U/ml penicillin and 100 mg/ml streptomycin, was used to continue the culture at D14, and cells were harvest at D16 (termed as rESC-RPC1) for analysis or transplantation. In the alternative differentiation method (longer suspension culture method), the suspension culture was maintained to D14 and the cells then digested for adherent culture to D16 for analysis or transplantation (termed as rESC-RPC2) (Fig. 1a).

### Live imaging of neuroectodermal sphere formation

The live images of the cultured cells were obtained using a Nikon BioStation IM-Q system with inverted microscopes (multi-photon) and a build-in CO_2_ incubator. About 30 rESC aggregates were suspended in 5 ml neuroectoderm differentiation medium with Matrigel (GFR) in a 6-cm low attachment Petri dish in the incubator. Photos of cell aggregates were obtained every 30 minutes from D1 to D8. Movies were exported from the Biostation IM software program.

### Immunofluorescence staining

Immunofluorescence staining, used to examine the post-transplantation behaviors of the donor cells and measure the thickness of the retinal outer nuclear layer (ONL), was performed as described previously [15]. Antibodies against the following proteins were used at the manufacturers’ recommended dilutions: Sox2 (Rabbit), Pax6 (Rabbit, Covance, Emeryville, CA, USA), Pax6 (Mouse, DSHB, Iowa City, IA, USA), Nestin (Mouse, BD Biosciences), BrdU (Rat, AbD Serotec, Kidlington, Oxford, UK), PCNA (Mouse, Sigma), Rax (Rabbit, Abcam, Cambridge, UK), Otx2 (Rabbit, Chemicon), Rhodopsin/Opsin (Mouse, Sigma-Aldrich), Recoverin (Rabbit, Millipore), GFAP (Rabbit, Abcam), Map2 (Mouse, Chemicon, Temecula, CA, USA), pH3 (Rabbit, Millipore), Bassoon (Mouse, ENZO Life Sciences, Farmingdale, NY, USA), Synaptophysin (Rabbit, Invitrogen, Camarillo, CA, USA), EGFP (Rabbit, Invitrogen), EGFP (Mouse, MBL, Naka-ku, Nagoya, Japan), GS (Rabbit, Sigma-Aldrich), Cralbp (Mouse, Abcam), Tuj1 (Mouse, Promega, Medison, WI, USA).

For secondary antibody staining, we used the corresponding Cy3 and fluorescein isothiocyanate (FITC) fluorescent-tagged antibodies (Jackson, West Grove, PA, USA or Proteintech, Chicago, IL, US). Nuclear staining was performed with 4′,6-diamidino-2-phenylindole (DAPI, Molecular Probes, Eugene, OR, USA). Stained sections were analyzed with a LSM710 confocal microscope (Zeiss, Jena, Germany).

In addition to the whole retinal examination, we focused more on the donor cells injection spots and the surrounding retina (see below for detail). The ONL thickness was measured based on DAPI staining and calculated as compared with the scale bar (μm).

### 5-Bromo-2′-deoxyuridine incorporation analysis

The cells were plated onto coverslips coated with PDL (Millipore) and Matrigel (BD Biosciences). 5-bromo-2′-deoxyuridine (BrdU, Sigma-Aldrich) was added at a final concentration of 10 μM. Four hours later, cells were fixed for further immunostaining assay.

### RT-PCR and qPCR

Total RNA was extracted from cultured cells or dissociated retina tissue using TRIzol® (Invitrogen) followed by chloroform extraction according to the manufacturer’s protocol. For RT-PCR analysis, 2 μg total RNA was reverse-transcribed using RevertAid™ M-MuLV RT kit (Fermentas, Hanover, MD, USA) and random hexamer primer according to the manufacturer’s instructions. PCR was performed on 1/20 of the final cDNA volume using the 2 × Taq PCR master mix (Promega). Reactions were performed at 60 °C for 30 cycles.

Real-time RT-qPCR was performed with FastStart Universal SYBR Green Master (ROX) (Roche, Mannheim, Germany) according to the manufacturer’s instructions. Signals were detected on an ABI PRISM 7900 machine (Applied Biosystems, Carlsbad, CA, USA). RT-qPCR was performed for various genes and results were normalized to GAPDH levels.

Sequences of primers used in this study are provided in Additional file 1: Table S1.

### Flow cytometric analysis

The cultured cells were dissociated by TrypLE (Gibco) into a single cell suspension, and then fixed with 4 % paraformaldehyde and permeabilized by ice-cold methanol. Before staining, the sample cells were washed and resuspended at 5 ~ 10 × 10^6^ cells/ml in PBS containing 1 % BSA and 0.03 % NaN_3_ (wash buffer) and then stained by the specific antibodies (PerCP-Cy™5.5 Mouse anti-Sox2, BD Biosciences; Alexa Fluor® 647 Mouse anti-GFAP, BD Biosciences; Alexa Fluor® 647 Mouse anti-Nestin, BD Biosciences; Alexa Fluor® 488 Mouse anti-Ki-67, BD Biosciences; PE anti-mouse CD133, Biolegend, San Diego, CA, USA). After antibody conjugate was added, the cells were incubated in the dark on ice for 30 min, and washed twice with wash buffer. The cell samples were resuspended and analyzed on a flow cytometer (Accuri C6, with the C6 software, BD Biosciences).

### Generation of knock-in rESC lines

The gene-targeting strategy and vector construction for Rax::EGFP is illustrated in Additional file 2: Figure S1. The site-specific transcription activator-like effector nucleases (TALENs) [55] were engineered to facilitate homologous recombination in rESCs. The TALENs (Forward target loci: CCTAGACACCTTTCCT; Reverse target loci: CCCGCTCCTTCGAGCC) designed to cause a double-strand break in exon1 of rat Rax gene and the targeting construct were constructed by ViewSolid Biotech Co. Ltd. (Beijing, China). To generate the targeting construct for homologous recombination in rESC, the 5′ arm (535 bp) and 3′ arm (808 bp) were amplified by PCR from rESC (DA8-16) genomic DNA. The cDNA of EGFP was fused in-frame into the first exon of *Rax* gene at the initial ATG. A PGK promoter-driven neomycin-resistance selection cassette flanked by loxP sites was inserted downstream of EGFP. The rESCs (DA8-16) were cotransfected with the targeting vector and both TALEN vectors using Fugene HD (Roche). Transfected cells were plated onto Neo-resistant feeder MEF cells. Homologous-recombinant rESCs (single clone) selected with 500 μg/ml G418 (Gibco) were picked and screened by PCR genotyping and further confirmed by Sanger sequencing. The floxed PGK-neo cassette was removed by transient transfection with the Cre-expressing plasmid (pCAG-GS-Cre) using FuGene HD and the resultant subclone Rax-G-DA#60 was established.

### Dissociation of the retinal tissue and in vitro culture

Primary retinal progenitor cells (P-RPC) from P1 Sprague–Dawley (SD) rats were digested in the papain dissociation system (Roche) as described previously [15]. The cells were then suspended in GMEM supplemented with DNase I (0.05 mg/ml) and kept on ice prior to virus conduction, RNA extraction or collected in TRIzol® for microarray analyses.

For EGFP labeling, P-RPC were plated on a Matrigel coated 10-cm dish and cultured in Neurobasal A® medium (Gibco) supplemented with 2 % B27 (Gibco), 20 ng/ml EGF (Peprotech, Rocky Hill, NJ, USA), 20 ng/ml bFGF (Peprotech), 0.1 mM nonessential amino acids, 1 mM glutamine (Gibco), 100 U/ml penicillin and 100 mg/ml streptomycin. Lentivirus carried EF1α promoter driven EGFP were prepared as described [15] and infected the plated P-RPC. After 48 hours, P-RPC were digested by TrypLE and resuspended in PBS supplemented with DNase I for transplantation.

### Animal experimentation

All animal experiments were conducted with the approval and under the supervision of the Animal Care Committee at the Shanghai Jiao Tong University School of Medicine. Animals were cared for in accordance with the Association of Research for Vision and Ophthalmology statement for the use of Animals in Ophthalmic and Vision Research.

Twenty one-day-old, tan-hooded and pink-eye congenic RCS rats, breeding under a 12 hr light/dark cycle, were used in this study. The left eyes (oculus sinister, OS) were treated with cell (2 × 10^5^ cells per eye in 2 μl PBS) transplantation while the right eyes (oculus dexter, OD) were injected with 2 μl PBS in the subretinal space to serve as sham control. RCS rats without subretinal injection were used as the disease control.

The rats were anesthetized and the subretinal transplantation was performed as described previously [14]. Briefly, under a stereo microscope, a 30-gauge needle was inserted into the vitreous chamber 1 mm behind the corneoscleral limbus, and vitreous fluid was drained off to reduce the intraocular pressure. Subsequently, a 33-gauge needle (Hamilton, Reno, NV, USA) carrying cells or PBS was inserted into the subretinal space, 1–2 mm temporal to the optic nerve papilla and between the two major retinal blood vessel branches. The eyes were examined at two, four and six weeks, as well as two months after the transplantation with various methods, such as electroretinographic (ERG) analysis, immunofluorescence staining, and so on.

### Electroretinographic analysis

Corneal ERG recordings from both eyes of rats were obtained at two, four and six weeks after the transplantation or sham injection, with an AVES system (Kanghua Rui Ming Technology Co. Ltd., Chongqing, China) following the procedures described previously [56].

### Microarray analysis

The microarray data are accessible at the GEO database under accession number GSE67213. RNA samples from three independent experiments were hybridized to a whole rat gene expression microarray (Affymetrix GeneChip® Rat Genome 230 2.0 Array) following the manufacturer’s instructions. For each sample, the background was removed, and raw data were normalized by MAS 5.0 algorithm, Gene Spring Software 11.0 (Agilent Technologies, Santa Clara, CA, USA). A hierarchical clustering of samples was performed using Cluster 3.0 software (Michael Eisen, Stanford University). Heat maps were generated using Java Treeview software. Gene ontology (GO) analyses were performed using DAVID 6.7 [57].

### Statistical analysis

All values were analyzed by Student’s *t* test or one-way analysis of variance (ANOVA) to determine the significance of the differences. *P* < 0.05 was considered statistically significant.

## Results

### In vitro differentiation of rESCs into RPCs

In order to develop an efficient retinal lineage differentiation protocol for rESCs, we tested some key factors reported on mouse and human ESC differentiation towards retinal lineage and their different combinations [17, 58, 59], and established the current optimized protocol (Fig. 1a). Briefly, we started with the SFEBq (quickly-aggregated/Serum-Free Embryonic Body) [60] culture by aggregating single rESCs in ultra-low-attachment 96-well plates (5,000 cells/well) with a serum-free differentiation medium (GMEM + 20 %KSR) containing a Wnt inhibitor (IWR-1e), which is known to have a rostralizing effect [61], to counteract the caudalizing activity of the high concentration of KnockOut™ Serum Replacement (KSR) during the early culture phase [17]. Matrigel (growth-factor-reduced, GFR) was added to the medium to make its final concentration 1 % on day 0. The cells were maintained under this condition until day 8, when a neuroectoderm like sphere structure appeared (Fig. 1b and Additional file 3: Movie S1). From day 8 to day 14, the differentiation medium was changed to GMEM containing 10 % KSR and 10 % FBS. At day 10, the aggregates were digested into single cells and passed into poly-D-lysine (PDL) and Matrigel coated plates for adherent culture. The culture medium was changed to N2 and DMEM/F12 containing 10 % FBS at day 14 and cells were harvested at day 16 (termed as rESC-RPC1). The tested donor cells were also prepared by an alternative method: the suspension culture was maintained to day 14, when aggregates were digested into single cells for adhesive culture and harvested on day 16 (termed as rESC-RPC2). For rESC-RPC2, cells were cultured in a GMEM differentiation medium containing 10 % KSR and 10 % FBS from day 8 to day 16 (Fig. 1a).

Since the neuroectoderm-like sphere structure is considered a critical stage for SFEBq self-organizing differentiation [17], we examined the aggregates for the neuroectoderm-like sphere structure by whole mount immunostaining at day 8. The cells in the neuroectoderm-like structure expressed neural progenitor cell (NPC) marker Sox2 and anterior neural marker Otx2 (Fig. 1c), suggesting that these rESCs had been differentiated into NPCs. In order to better monitor the process of rESC differentiation into retinal lineages, we generated a rESC line (Rax::EGFP rESC) carrying a EGFP reporter inserted homologously into one allele of the genomic locus of *Rax*, a marker for the retinal progenitors, using the TALEN technique (see Methods and Additional file 2: Figure S1). The Rax::EGFP rESCs were induced to retinal lineage differentiation by the same SFEBq method as used for rESC-RPC2 generation. Rax-driven expression of EGFP could be detected in some cells in the neuroectoderm-like structure at day 8, indicating the retinal lineage differentiation (Fig. 1d). Cryosection immunofluorescence examination revealed that cells in the Rax::EGFP rESC-derived neuroectoderm-like structure expressed NPC markers, including Pax6, N-Cadherin and Nestin (Fig. 1e). Furthermore, it appeared that E-Cadherin positive cells were negative for Rax::EGFP but N-Cadherin positive cells and Nestin positive cells were partially colocalized with Rax::EGFP cells. Although Pax6-positive and Rax::EGFP-positive cells were partially overlapped, all Rax::EGFP cells expressed Rax. These findings verified that Rax::EGFP authentically represented the endogenous expression of Rax in rESC-derived cells. In addition, we examined the expression pattern of pluripotency-associated markers and neural or retinal lineage markers during the differentiation process by RT-PCR assays (Fig. 1f and Additional file 4: Figure S2a). The results showed that pluripotency markers Oct4 and Nanog were down regulated upon differentiation while the eye field transcription factors (EFTFs), such as *Pax6*, *Rax*, *Six3*, *Six6,* and *Lhx2* were up regulated, suggesting the retinal lineage differentiation. Consistent with the immunofluorescence staining result, the transcript of *Rax* was detected at day 8 and afterwards. The neuronal marker *Tuj1* began to express in the early days and became upregulated in the late stage of differentiation, while the glia marker *GFAP* could only be detected late during differentiation. For an unknown reason, *Tuj1* expression at D5 was relative weak in both rESC-RPCs. Generally, both rESC-RPCs showed similar differentiation patterns although retinal lineage gene expressions were a little weaker in rESC-RPC1 than rESC-RPC2 (Fig. 1f and Additional file 4: Figure S2a). Moreover, expression of the photoreceptor precursor marker *Recoverin* was also found in rESC-RPC2 rather than rESC-RPC1 by the end of differentiation (Fig. 1f). Expression patterns of these markers suggest that rESC-derived cells were comprised primarily of RPCs with some NPCs and photoreceptors.

### Characterization of rESC-derived RPCs

**Fig. 2:**
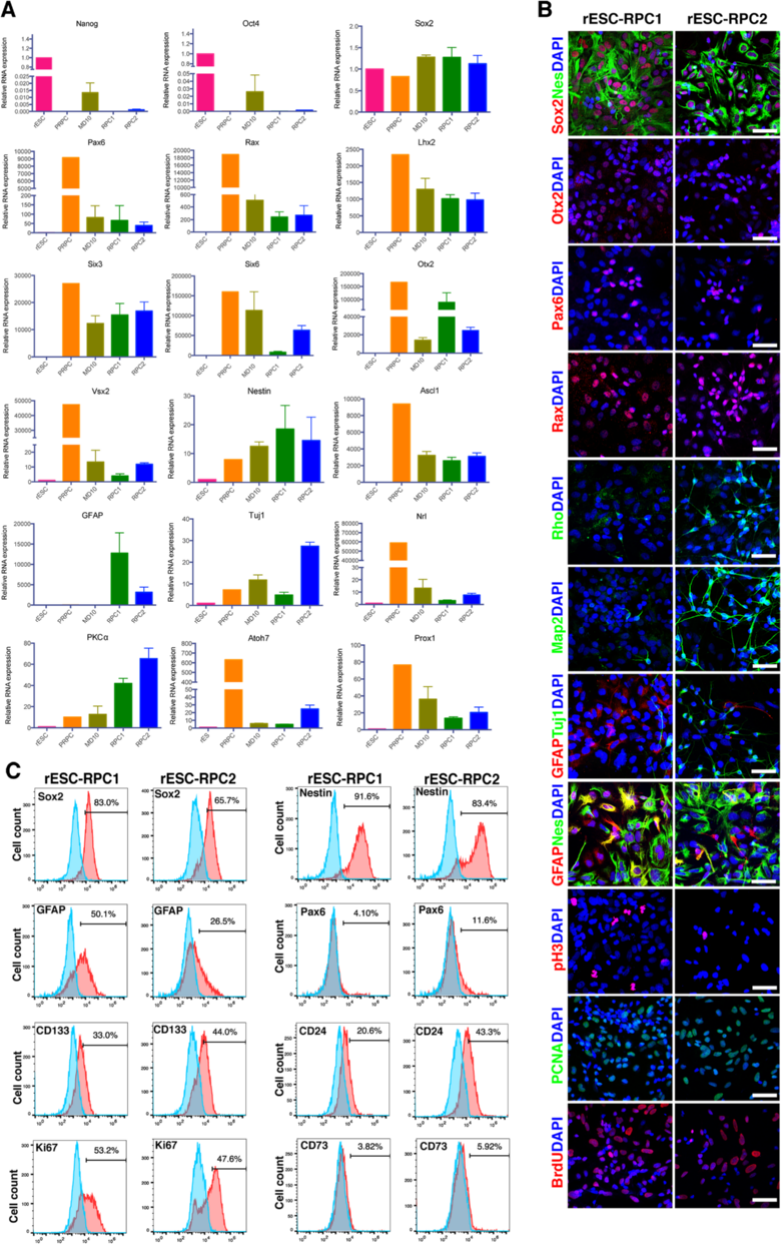
Characterization of rESC-RPCs at D16. **a** Comparisons of various marker expressions among rESCs, rESC-RPCs and other controls, including samples of differentiating rESCs at day 10 and P-RPCs. The relative mRNA levels of marker genes were determined by RT-qPCRs. Error bars represent the mean ± SD, n = 3. **b** Representative confocal immunofluorescence images of rESC-RPCs for NPC markers (Sox2, Nestin/Nes), anterior neural marker (Otx2), proliferation markers (pH3, PCNA and BrdU), retinal progenitor markers (Rax and Pax6), neuronal marker (Tuj1) and astrocyte and radial glia cell marker (GFAP). Scale bar: 50 μm. **c** Representative FCM profiles of rESC-RPCs for subpopulations expressing Sox2, Nestin, CD133, GFAP, Ki67, CD73 and CD24. Corresponding IgG was applied as the negative isotype control. *rESC* rat embryonic stem cell, *RPCs* retinal progenitor cells, *RT-qPCR* reverse transcription-quantitative polymerase chain reaction, *NPC* neural progenitor cell

To learn more about the rESC-RPCs harvested on day 16 of the differentiation at a molecular level, we compared the expression pattern of various markers between undifferentiated rESCs and rESC-RPCs (both rESC-RPC1 and rESC-RPC2). The P-RPCs prepared from postnatal day 1 (P1) Sprague–Dawley (SD) rats were used as the control. Differentiated rESCs at day 10 were also included (Fig. 2a). The RT-qPCR results showed that pluripotency-associated genes (*Nanog* and *Oct4*) were almost not detectable in both rESC-RPCs and P-RPCs, whereas the expressions of NPC markers (*Sox2*, *Nestin*, and *Ascl1*), anterior neuroepithelium progenitor marker *Otx2*, as well as neuronal marker *Tuj1* were higher in rESC-RPCs and P-RPCs than in rESCs. Moreover, EFTFs, including *Rax*, *Pax6*, *Lhx2*, *Six3*, and *Six6* [62], were induced to express in rESC-derived cells, even though at lower levels when compared with P-RPCs. Furthermore, the expression of the neural retina progenitor marker *Vsx2* (*Chx10*), specific bipolar cell marker *PKCα* and rod photoreceptor precursor marker *Nrl* were also detected in the rESC-RPCs. The difference between rESC-RPC1 and rESC-RPC2 is that rESC-RPC1 expressed a higher level of *GFAP*, whereas rESC-RPC2 had a higher level of *Tuj1*.

To further characterize rESC-RPCs, we examined their protein expression of neural lineage and retinal progenitor markers with immunofluorescence staining (Fig. 2b). Extensive staining of Sox2 and Nestin was readily detected in both rESC-RPC1 and rESC-RPC2. Retinal progenitor markers Pax6, Otx2, and Rax were all expressed in these two cell populations, but the staining for Otx2 is much stronger in rESC-RPC1 than that in rESC-RPC2 while the staining for Pax6 and Rax were similar in both. Consistent with the RT-qPCR results, there were more GFAP-expressing cells in rESC-RPC1 than in rESC-RPC2, while rESC-RPC2 had a higher fraction of cells expressing Tuj1 and Map2 than rESC-RPC1, confirming that rESC-RPC2 contained more neurons while rESC-RPC1 had more glia cells. Double staining of GFAP and Nestin revealed that a considerable part of the GFAP positive cells in rESC-RPC1 were Nestin-positive radial glia neural progenitor cells. In particular, rESC-RPC2 contained more cells expressing rod photoreceptor marker Rhodopsin/Rho than rESC-RPC1. Furthermore, similar proliferation capacity was verified by positive staining for antibodies against pH3 and PCNA as well as incorporation of BrdU into both groups of rESC-RPCs.

Finally, as shown in Fig. 2c, we analyzed the cellular component of rESC-RPCs with flow cytometry (FCM). The majority of rESC-RPCs were both Sox2-positive and Nestin-positive, indicating they were differentiating towards retinal neurons rather than RPE. About one third of rESC-RPCs were reactive to the antibody of CD133, which stains neuroblastic embryonic retina and in the developing photoreceptor cell layer during postnatal development [63]. Furthermore, some rESC-RPCs were reactive to CD73 antibody, and a higher fraction of rESC-RPC2 (about 40 %) was reactive to the antibody to CD24 than that of rESC-RPC1 (about 20 %). CD24 has been reported ubiquitously expressing throughout the developing embryonic and postnatal neural retina during retinal development [63]. These two markers, CD73 and CD24, have been used for sorting photoreceptors for transplantation [63]. The low level of CD73 expression might indicate the prenatal stage of rESC-RPCs as compared with the in vivo developmental stage [63]. A similar percentage (about 50 %) of Ki67 positive proliferating cells was found in the two rESC-RPCs. Consistent with the immunofluorescence staining result (Fig. 2b), we observed more GFAP positive cells in rESC-RPC1 (50 %) than in rESC-RPC2 (26.5 %) (Fig. 2c). Based on these data, we concluded that rESC-RPCs achieved substantial retinal lineage differentiation by day 16, and such rESC-RPCs contain committed retina lineage cells and some NPCs.

### Whole genome gene expression analysis of rESC-RPCs

**Fig. 3:**
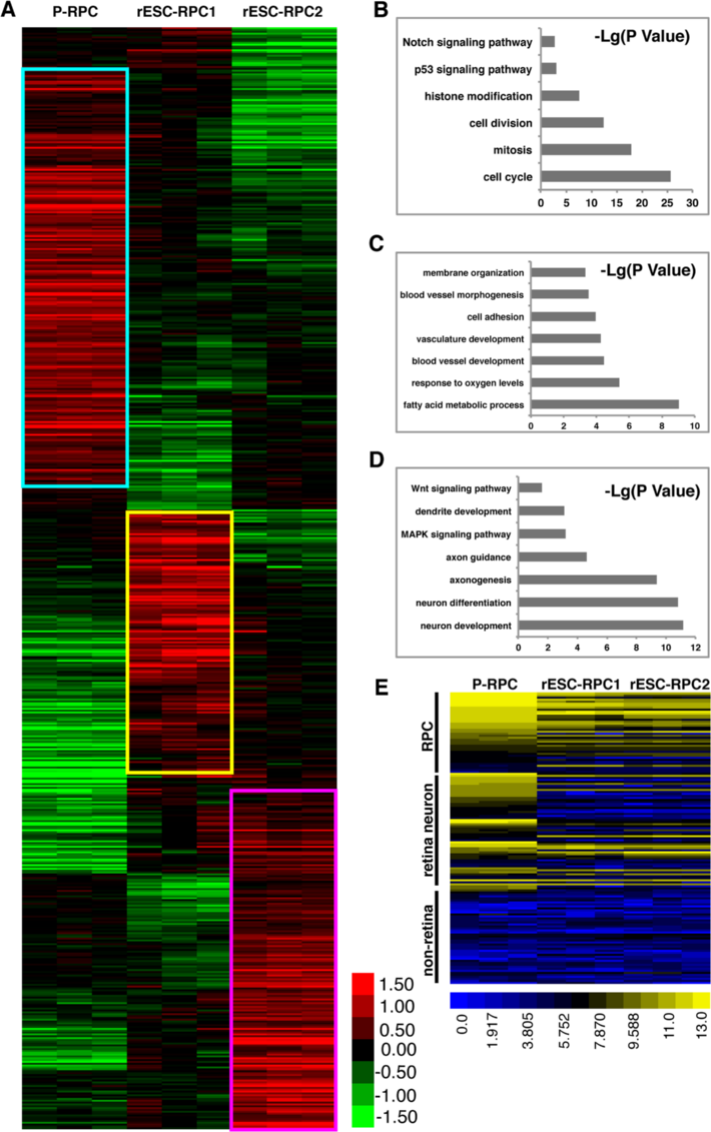
Microarray analysis of gene expression of rESC-RPC1, rESC-RPC2 and P-RPCs. **a** Heat map analysis of differentially expressed genes among rESC-RPC1, rESC-RPC2 and P-RPC. **b** GO analysis of highly expressed genes in P-PRC. Bar graph showing significance of enrichment terms for sets of genes in the *blue box* in **a. c** GO analysis of highly expressed genes in rESC-RPC1. Bar graph showing significance of enrichment terms for sets of genes in the *yellow box* in **a. d** GO analysis of highly expressed genes in rESC-RPC2. Bar graph showing significance of enrichment terms for sets of genes in the *magenta box* in **a**. **e** Heat map analysis of various genes expressed by RPC, retinal neurons and non-retinal tissues as listed in Additional file 5: Table S2. Log2 expression levels of the genes are shown in a blue-black-yellow gradient. *rESC-RPCs* rat embryonic stem cell-derived retinal progenitor cells, *P-RPCs* primary retinal progenitor cells, *GO* gene ontology

To further define rESC-RPCs at a global scale, we performed genome-wide transcript profiling for rESC-RPCs and P-RPCs with three biological replicates for each cell type. In total, 3,343 genes were differently expressed between rESC-RPC1 and P-RPC, 3,758 genes between rESC-RPC2 and P-RPC, and 2,045 genes between rESC-RPC2 and rESC-RPC1 at the level of two-fold and more (*P* < 0.05) (Table 1). We analyzed high expression genes of different groups according to the heat map (Fig. 3a). Gene ontology (GO) analysis showed that the highly expressed genes in P-RPCs primarily encoded molecules associated with cell cycle and mitosis (Fig. 3b). The genes highly expressed in rESC-RPC1 mainly encoded molecules associated with cell adhesion, blood vessel and vasculature development (Fig. 3c), while the genes upregulated in rESC-RPC2 predominantly associated with neuron differentiation, neuron development and axonogenesis (Fig. 3d) [57]. Analysis of the expression of genes in specific regions (Fig. 3e) showed that rESC-RPC1 and rESC-RPC2 had a similar expression pattern as RPC specific genes but almost no expression of non-retinal specific genes (non-retinal CNS genes, hepatic genes, cardiac genes, renal genes and pineal gland genes, Additional file 5: Table S2) [64], indicating the retinal lineage differentiation of RPC1 and RPC2. These results confirmed that these three kinds of cells had distinct features of the transcriptional signature of RPCs. What accorded with the results of marker gene expression was that rESC-RPC2 might have functions closely associated with differentiated neurons. It is also clear that rESC-RPCs and P-RPC share many characteristics and functions, even though they are different from each other in terms of whole genome gene expression.

**Table 1:** Numbers of changed genes among rESC-RPCs and P-RPCs

Comparison	Numbers of changed genes	Up-regulated	Down-regulated
RPC1 vs P-RPC	3343	1698	1645
RPC2 vs P-RPC	3758	1772	1986
RPC2 vs RPC1	2045	994	1101

*rESC-RPCs* rat embryonic stem cells-retinal precursor cells, *P-RPCs* primary retinal progenitor cells

*P* < 0.05, fold change ≥ 2

### The rESC-RPCs preserve the visual function in rats with retinal degeneration

**Fig. 4:**
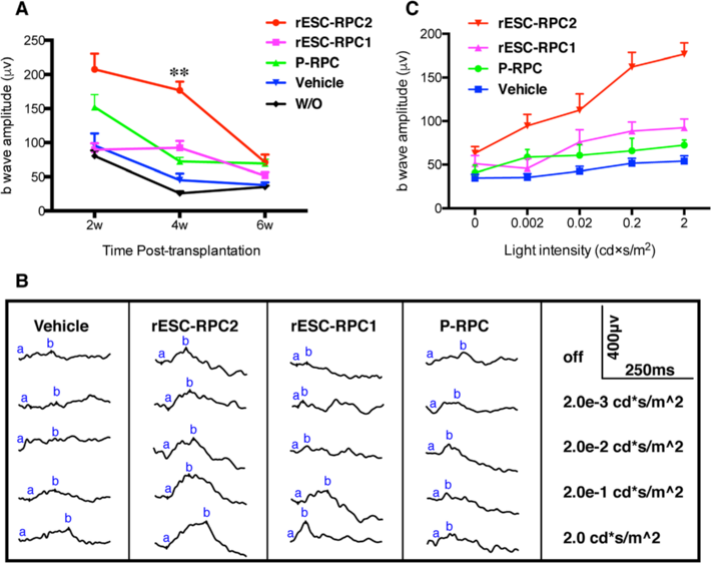
Visual function evaluation of the RCS rats after the transplantation of rESC-RPCs and P-RPCs. **a** ERG b-wave of the RCS rats at different time points after different treatments. Error bars represent the mean ± SEM, ANOVA, **P < 0.01 (rESC-RPC2 vs rESC-RPC1), n = 7 for two weeks and four weeks, n = 6 for six weeks. **b** Typical ERG waves of the RCS rats at four-week time point after the transplantation. **c** ERG b-wave of the RCS rats four weeks after rESC-RPCs and P-RPC transplantation at different light intensity stimuli. Error bars represent the mean ± SEM, n = 7 for each group. *RCS* Royal College of Surgeons, *rESC-RPCs* rat embryonic stem cell-derived retinal progenitor cells, *P-RPCs* primary retinal progenitor cells, *ERG* electroretinogram, *W/O* without treatment

To evaluate the therapeutic potential of rESC-RPCs, the cells were injected into the subretinal space of RCS rats. RCS rats received unilateral injection of the cells at P21 and the contralateral eyes were injected with vehicle as control for each animal. Meanwhile, transplantation of P-RPCs was performed in a group of RCS rats as positive control, since such cells had been proven to protect the retina in mouse with retinal degeneration [14, 15]. RCS rats without any treatment were used as the disease control. Scotopic electroretinogram (ERG) examination was conducted at two weeks (P35), four weeks (P49) and six weeks (P56) after the transplantation to evaluate the functional responses of photoreceptors and related retinal cells upon light stimulus. The ERG b-wave amplitude was used as the major parameter to evaluate rat visual function. The results showed that rESC-RPC2 significantly protected the visual function from degeneration at the early stage of treatment (Fig. 4a), while rESC-RPC1 only improved the function at four weeks, when compared with the vehicle control. On the other hand, in the positive control group, the transplantation of P-RPCs significantly improved the visual function only at two weeks and then lost the effect quickly. Typical ERG examination was also performed four weeks after the transplantations (Fig. 4b) and the b-wave responses in rESC-RPC2 treated rats were found to increase with the increase of light intensities (Fig. 4c). Thus, rESC-RPCs, especially rESC-RPC2, have potential as a candidate cell source for the treatment of retinal degeneration. However, almost no therapeutic effect was maintained beyond six weeks after the transplantation, even for the rESC-RPC2.

### Transplanted rESC-RPCs survive and migrate into the retina

**Fig. 5:**
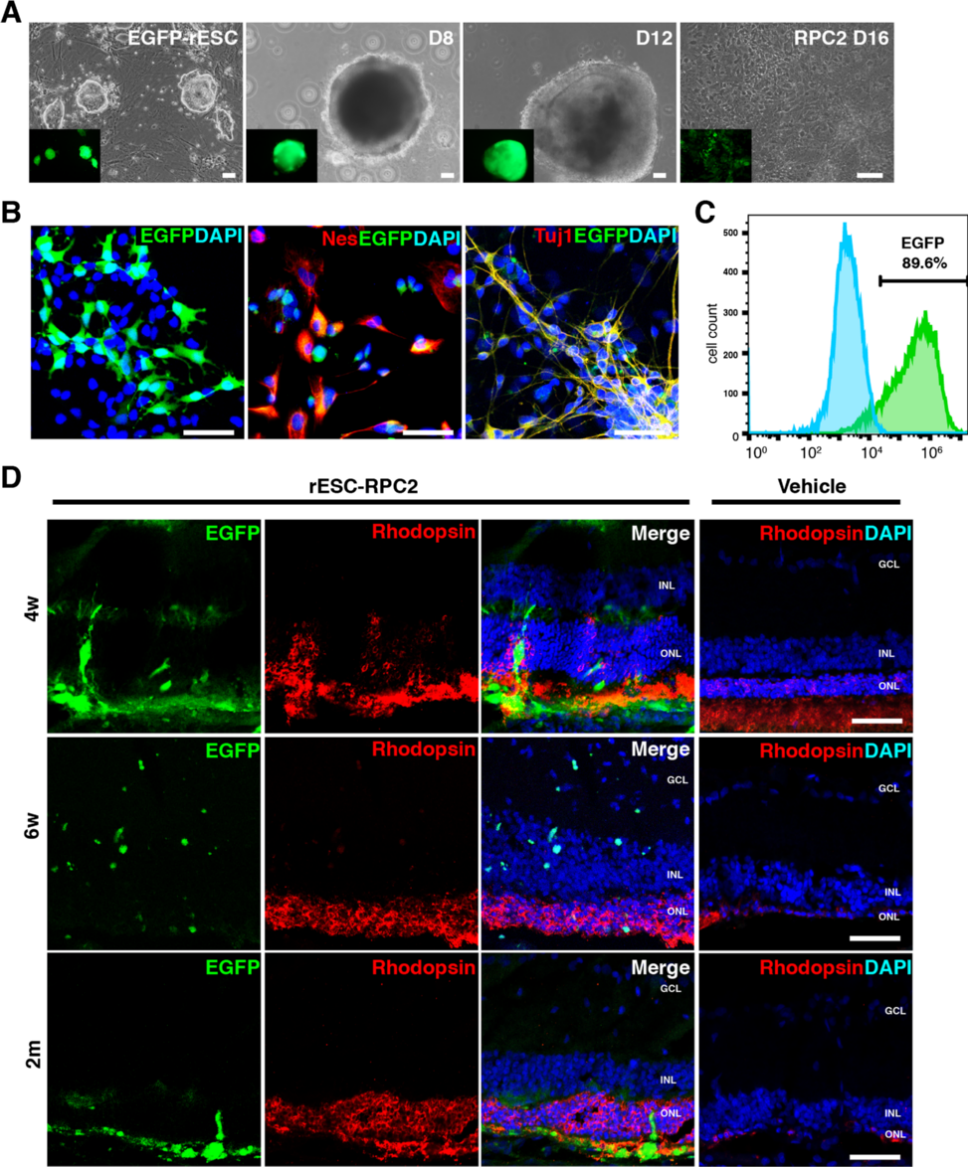
Subretinal transplantation of EGFP labeled rESC-RPCs in the RCS model. **a** Generation of rESC-RPC2 from EF1α::EGFP lentivirus labeled rESCs. Scale bars: 50 μm. **b** Immunofluorescence staining of EGFP-rESC-RPC2 at D16 before transplantation. Antibody against EGFP (*green*) and antibodies against Nestin/Nes and Tuj1 (*red*) were used. DAPI (*blue*) was used to highlight the nuclei. Scale bars: 50 μm. **c** Flow cytometric analysis of EGFP-rESC-RPC2 in comparison with unlabeled rESC-RPC2. **d** Grafted rESC-RPC2 could be detected in the retina at different times after transplantation. *GCL* ganglion cell layer, *INL* inner nuclear layer, *ONL* outer nuclear layer. Scale bar: 50 μm. *EGFP* enhanced green fluorescent protein, *rESC* rat embryonic stem cell, *RPC* retinal progenitor cell, *RCS* Royal College of Surgeons, *DAPI* 4′,6-diamidino-2-phenylindole

In order to trace the grafted cells, we labeled rESCs with EGFP by EF1α::EGFP lentiviral transduction (EGFP-rESCs) and differentiated them into RPCs following the same protocol described earlier in the study (Fig. 5a). As EGFP was constitutively expressed in cells at any stage, EGFP-rESC-RPCs could be detected before and after the transplantation (Fig. 5b and Additional file 4: Figure S2b-c). Immunofluorescence staining of EGFP-rESC-RPC2 displayed the co-expression of EGFP and Nestin or EGFP and Tuj1 (Fig. 5b). FCM analysis showed that about 90 % of rESC-RPC2 expressed EGFP before injection (Fig. 5c). To investigate whether the transplanted EGFP-rESC-RPCs could survive within the host retinal circumstance and even migrate into the inner retinal part, we performed immunofluorescence analysis at three time points, four weeks, six weeks and two months, after the transplantation (Fig. 5d). EGFP labeled donor cells could be detected at all the time points in the subretinal space, the ONL and the inner layers of the host retina, indicating the survival and migration of grafted cells in the retinas of RCS rats.

### EGFP-rESC-RPCs differentiate and integrate with host retinal cells and preserve the retinal structure

**Fig. 6:**
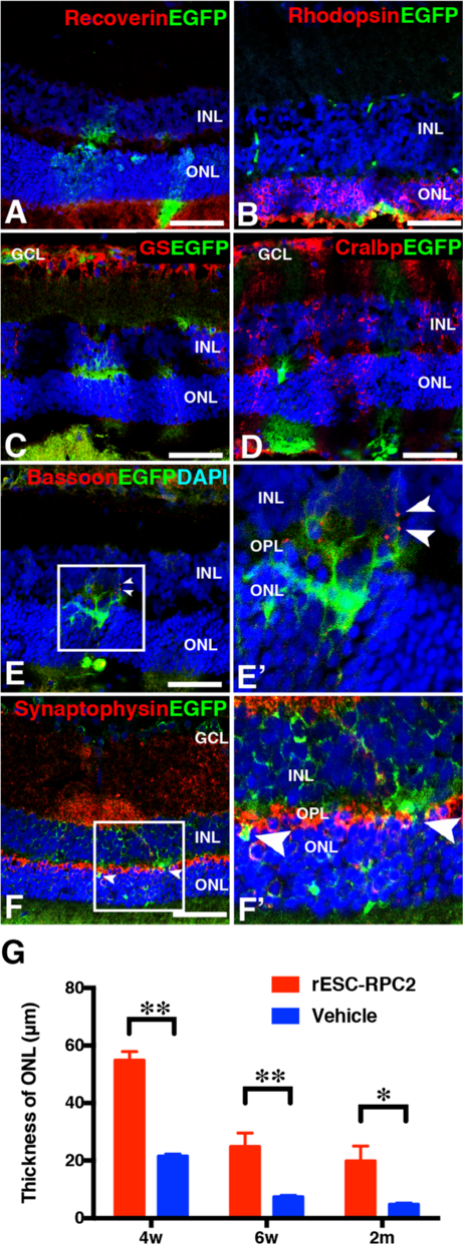
Differentiated EGFP-rESC-RPC2 integrate with host retina and preserve the retinal structure 4w after transplantation. **a**-**d** Differentiation of rESC-RPC2 into various retinal cells. Antibody against EGFP (*green*) and antibodies against retinal cell markers (*red*) were used for immunofluorescence staining. DAPI (*blue*) was used to highlight the nuclei. *GCL* ganglion cell layer, *INL* inner nuclear layer, *ONL* outer nuclear layer. Scale bars: 50 μm. **e** and **f** Integration of EGFP-rESC-RPC2 with retinal neurons. Colocalization of presynaptic markers Bassoon and Synaptophysin with EGFP expressed by donor cells in OPL was confirmed with immunostaining. *GCL* ganglion cell layer, *INL* inner nuclear layer, *ONL* outer nuclear layer. Scale bars: 50 μm. ***e'*** and ***f'***. The magnified images of the rectangles in **e** and **f**. *Arrow heads* indicate the colocalization of EGFP and Bassoon or Synaptophysin. *GCL* ganglion cell layer, *INL* inner nuclear layer, *OPL* outer plexiform layer, *ONL* outer nuclear layer. **g** Preservation of the retinal structure by EGFP-rESC-RPC2 transplantation. The thickness of ONL of rESC-RPC2 treated eye was compared with that of vehicle (PBS) injected retina. Data are shown as mean ± SEM, *t* test, *P < 0.05 and **P < 0.01, n = 6 (rats) for each group. *EGFP* enhanced green fluorescent protein, *rESC* rat embryonic stem cell, *RPC* retinal progenitor cell, *DAPI* 4′,6-diamidino-2-phenylindole

We next investigated if the transplanted EGFP-rESC-RPC2s could further differentiate into retinal cell types in the host retina. Fluorescence staining of samples from the four-week time point after the transplantation revealed that EGFP was co-stained with the photoreceptor precursor marker Recoverin (Fig. 6a) and the rod photoreceptor marker Rhodopsin (Fig. 6b), supporting the possibility that some EGFP-rESC-RPC2 could differentiate into photoreceptors after grafting into degenerative retinas. Such integration was also observed at two weeks and six weeks after the transplantation (Additional file 4: Figure S2d-g). However, we did not observe the co-expression of Müller cell markers (GS and Cralbp) (Fig. 6c and d) with EGFP. To further confirm the integration of transplanted EGFP-rESC-RPC2 within the host retina, immunofluorescence staining using antibodies against presynaptic markers (Bassoon and Synaptophysin) was conducted. We observed pronounced co-localization of the synaptic markers and the EGFP labeled donor cells (Fig. 6e-f and Additional file 4: Figure S2d-g) at the outer plexiform layer (OPL). These results made it clear that grafted cells formed new synapses with the host retina cells, providing the cellular basis for rESC-RPCs to functionally recover recipient retinas.

Finally, we examined whether the retinal structure could be preserved by the transplanted cells. The thickness of ONL was measured to reflect the general structure of the outer layer of the retina in this study. As showed in Fig. 6g and Additional file 4: Figure S2h, EGFP-rESC-RPC2 transplantation significantly preserved retinal structure and maintained the ONL thickness at 60 μm, while the ONL thickness reduced to 20 μm in untreated RCS rats, when examined at four weeks after the transplantation. It strongly supported the observation that rESC-RPC2 evidently improved the visual function of RCS rats at that time point. This trend lasted up to two months after the transplantation (Fig. 6g). Our data also indicated that rESC-RPC1 treatment and P-RPC treatment also provided protection to the retinas of RCS rats, but rESC-RPC2 provided stronger and longer protection to such retinal degeneration (Figs. 4a and c, 6g and Additional file 4: Figure S2h).

## Discussion

The laboratory rat (*Rattus norvegicus*) was the first mammalian species used for scientific study [65], and has been serving as one of the most widely used model animals for studying various human diseases, such as cell transplantation [45, 66], diabetic retinopathy [47], hypertension [46] and nerve regeneration [67, 68], as it is more relevant to humans than mice [33]. In addition, rat models are also employed as routine preclinical models for drug development [69, 70], gene therapy [71] and cell therapy [54]. Recently, rats have been demonstrated to share many common loci with humans, indicating the presence of shared pathways in rats and humans [72]. However, investigation of genetic manipulation in rat is extremely lagging behind mouse, as shown by the fact that the generation of rat ESCs fell almost three decades behind mouse [34, 36, 37]. Here, using an established rat ESC cell line [38], we developed a RPC differentiation method and demonstrated that rESC-derived RPCs could rescue the degenerated retina in RCS rats. This finding not only paves the way for using rat ESCs to study mammalian development and regenerative medicine but also for pharmacological testing and drug screening in culture dishes.

Previous studies proved that epiblast cells in rat had the ability to generate hypoblast in contrast to those in mouse [43], implying the intrinsically different capacity between mouse ESC and rat ESC. Researchers found that it was difficult for rESC to form embryoid body (EB), a critical stage for *in vitro* differentiation, and most of the cells died in the initial stage of EB formation [37–40]. They suggested that EB formation of rESCs needed a specific environment which could be achieved by combining the conditional medium collected from MEF cultures with 2i, PD0325901 and CHIR99021, and the Rho-associated kinase inhibitor Y27632, to efficiently support the formation and survival of EBs. Following these methods, some studies generated tripotent neural progenitor cells from rESCs [40]. In this study, we modified Sasai’s method which combined SFEBq with Matrigel culture [17] to directly differentiate rESCs to RPCs instead of using conditional medium and 2i, even without Y27632. Under this culture condition, the rESCs could differentiate into uniform neuroectodermal spheres efficiently in an ultra-low-attachment U96-plate system, similar to the hanging-drop method [38]. The rESCs could hardly form a neuroectodermal sphere in a low KSR concentration medium (1.5 % ~ 2 %) as used for mouse retinal differentiation (data not show), but could proceed with retinal differentiation under the hESC differentiation condition (20 % KSR and Wnt inhibitor). Following our modified methods, we obtained RPCs derived from rESCs and these rESC-RPCs highly expressed key eye field transcription factors (EFTFs) like Lhx2, Pax6, Six3 and Six6, and RPC markers (Rax, Otx2 and Vsx2) (Figs. 1f and 2), similar to mouse ESC derived RPC [15] and human ESC derived RPC [73]. The genome-wide gene expression assay also showed that the two kinds of rESC-RPCs and the primary RPCs (P-RPCs) highly expressed RPC related genes (Fig. 3e), but their expressions of non-retinal genes (non-retinal CNS genes, hepatic genes, cardiac genes, renal genes and pineal gland genes) were all very low (Fig. 3e). On the other hand, the term RPCs is used for all the cells in the development of the retina between retinal stem cells and all types of retinal cells, including retinal neurons, Müller glia cells as well as RPE cells, and are reported to contain a pool of multipotent cells dependent on different developmental stages and position in vivo [74–77]. So, RPCs contain different retinal lineage markers at different development stages. In addition, we also detected the expression of the rod photoreceptor marker Rhodopsin in these rESC-RPCs. These data demonstrate clearly that rESC can be directed into a retinal fate and generate RPCs. This provides a new system to study and treat degenerative retinal diseases.

In this study, we also employed two different methods to differentiate rESCs into retinal lineage cells. Both methods share the neuroectodermal induction process but, thereafter, one group of cells was switched to culture on Matrigel-coated plates at D10 (rESC-RPC1), while the other cells remained in suspension culture until D14 (rESC-RPC2). The rESC-RPC1 tends to express the glia cell marker GFAP while rESC-RPC2 tends to express the neuronal marker Tuj1 and Map2 (Fig. 2). That is to say, early and longer adherent culture of NPC promotes glia cell differentiation under the serum condition while longer suspension culture gives rise to neuronal differentiation. A recent study also mentioned a similar concept that murine RPC cultured under a high concentration of FBS (10 % ~ 20 %) increased GFAP positive cells and decreased neurons [78]. In the transplantation study, we further confirmed that the rESC-RPC2 was more suitable than rESC-RPC1 for treating retinal degeneration, because engraftment of rESC-RPC2 significantly preserved vision better than rESC-RPC1 (Fig. 4). These results imply that neuronal lineage cells instead of glia cells are favorable for visual function preservation in the early stage of the degeneration in RCS rat. We speculated that the mechanism might be that the engrafted rESC-RPC2 integrated into the host retina, as observed at two, four and six weeks or even at two months after the transplantation, and established communication directly with recipient retinal neurons (Fig. 6e-f and Additional file 4: Figure S2d-g) by replacement of degenerated photoreceptors. This was also confirmed by the observation of the presynaptic markers (Bassoon and Snaptophysin) colocalization between the donor cells and the host retina, similar to what is observed in mouse and human ESC-derived cells [22, 79].

The RCS rat, an animal model of retinal degeneration, carries a *Mertk* gene mutation. Loss of this gene leads to the failure of RPE cells in RCS rat to phagocyte shed outer segment of photoreceptors, which is a critical function of normal RPE cells. The accumulation of outer segment debris causes the degeneration and death of photoreceptors, which are directly related to the loss of vision. So, the RCS rat is considered a good model to test the therapeutic effects of donor cells for rescuing photoreceptors and RPE cells. In a relatively earlier stage of retinal degeneration, RPE cells degenerated and photoreceptors were degenerating, and, therefore, transplantation of RPE cells might be a proper therapy. They could replace the dysfunctional RPE cells then improve the environment for photoreceptors to function well. However, at a relatively later stage when photoreceptors were dead, since photoreceptors as terminally differentiated neurons could not regenerate and proliferate, vision could only be improved when the dead photoreceptors are replaced by the transplanted photoreceptors or the transplanted cells derived photoreceptors, but not RPE cells. In such cases, the cells that can differentiate into both photoreceptors and RPE cells are ideal donor cells. Based on such expectations, rESC-RPCs were used in this study, which expressed key EFTFs and might be able to generate both photoreceptors and RPE cells after transplantation. The transplanted cells, especially the rESC-RPC2, improved the rat vision for several weeks. However, six weeks after the transplantation, when the RCS rats were nine weeks after birth and their retinal degeneration was more severe, the engrafted cells failed to rescue the recipient retina (Fig. 4a) and the protective effect of rESC-RPC2 declined [80]. Considering our observations that rESC-RPC2 differentiated into functional neurons but not RPE cells after transplantation, we believe that the transplanted cells rescued rat vision by differentiating into photoreceptors in the early stage but failed to maintain their effects because the RPE’s function was not rescued. As we didn't correct the gene mutation in the RPE cells, outer segment debris accumulation and other related changes remained, so that transplanted cells-derived healthier photoreceptors and the vision could not last long.

It is worth mentioning that the visual function of the RCS rats was evaluated by ERG, a commonly used objective evaluation for visual function in experimental animals [81–83], and for evaluation of the therapeutic effect of various cell transplantation experiments in various animals with retinal degenerative [9, 15, 56, 84, 85]. It is based on the principle that activities of retinal neurons could be detected around the eye. The major parameter of the examination, the b-wave, was significantly improved after the rats were treated with rESC-RPCs, reflecting the improved visual function. In fact, the b-wave is not a direct reflection of photoreceptors but its amplitude correlates directly with the activity of bipolar cells [86]. However, bipolar cells are post-synaptic to [87], and activated by, the photoreceptors. In the ERG examination, light is the only stimulation and the photoreceptors are the only cells of our body which respond to light stimulation. So, an activated bipolar response indicates the existence of functional photoreceptors connecting to the bipolar cells. Degenerated photoreceptors, such as those in the RCS rat, activated fewer bipolar cells and yielded lower b-wave amplitudes, and the b-waves extinguished with progression of the degeneration, even though the bipolar cells remained in the inner nuclear layer [88]. On the other hand, the b-wave should be improved if the donor cells could preserve or improve the function of photoreceptors.

Preservation of retinal structure is another major consideration for evaluating a cell therapy, especially in RCS rats, which suffer from degeneration in the retina and experience rapid decrease of the ONL layer [89, 90]. Therefore, measurement of the ONL thickness has been widely used for assessment of the intervention effects, regardless of the drug treatment, cell transplantation or gene therapy, in the RCS rat retina [45, 91–95]. RPE cells, including RPE cell lines [45, 96] and human ESCs-derived RPE cells [9, 54], have been well proven to be protective for retina in the RCS rats. Some other neural lineage cells, such as cortical neural progenitor cells [84, 97], central nervous system stem cells [98, 99] and RPCs [80], were also reported to be protective to retina in this model. Several mechanisms may be involved in such stem cell intervention. For example, some transplanted cells might protect the retina by releasing trophic factors [80, 84], while others, such as hNPC^CTX^, could form an extra RPE-like layer [97] which reduced the debris of photoreceptor outer segments even though it did not express RPE marker. As shown in Fig. 5d, a similar cell layer formed by EGFP expressed cells was observed in this study. Replacement of the degenerated cells by donor cells should be an ideal mechanism because the retinal structure would be repaired as well. It was reported to be true in the case of the transplantation of human neural stem cells into RCS rats, which phagocytosed the photoreceptor outer segment as RPE cells did in normal rats [99]. However, other studies reported that transplanted human RPCs failed to express retinal markers and expressed the progenitor cell marker Nestin even though the donor cells distributed in multiple neural retinal layers [80]. In this study, we observed that the grafted rESC-RPC2 colabelled with photoreceptor markers including Rodopsin and Recoverin (Fig. 6a-b and Additional file 4: Figure S2b-c), indicating further differentiation and integration of the transplanted cells. It might be true that most of the transplanted neural cells could not integrate into the host retina or differentiate into retinal cells [14, 80, 84], but a relatively small number of transplanted progenitor/retinal cells could be sufficient to rescue visual function [8]. As for the retinal structure reconstruction, we observed that the thickness of the ONL was also well maintained in the rESC-RPC2 transplanted group, even a small fraction of donor cells were detected there (Fig. 6g and Additional file 4: Figure S2h). As for the stronger protective effects of rESC-RPC2 than rESC-RPC1 and P-RPC, our understanding is that rESC-RPC2 contains more differentiated retinal neurons including some photoreceptors than rESC-RPC1 and P-RPC, so that more degenerated retinal cells were replaced. From the point of view of development, rESC-RPC2 may also represent an earlier stage than P-RPC in the development of RPCs towards retinal neurons and, thus, they had better potential for differentiating into retinal neurons and photoreceptors for the replacement. The different animal models and the donor cells from different species might be another reason causing the difference. A good intervention of P-RPCs in previous studies was obtained from sodium iodate-induced retinal degeneration in mice [15], and the situation in rats here could be different.

Up to two months after rESC-RPC2 transplantation, no obvious abnormal cell proliferation was observed.

## Conclusions

This study provides a new and practical method for neural and retinal differentiation of rat ESCs, which could be used in a wide range of biomedical and translational research, including pathogenesis, drug discovery, toxicology, gene therapy technology, and regenerative medicine. Moreover, this study proved that rESC-derived RPCs, especially the retinal neuron-enriched rESC-RPC2 obtained through the longer suspension culture method, could effectively protect the retinal structure and function in RCS rats.

## Additional files

Additional file 1: Table S1. PCR primer sequences. (DOC 53 kb) Additional file 2: Figure S1. Construction of Rax::EGFP reporter rESCs. **a** Knock-in strategy for the Rax::EGFP reporter construct. *Red arrows*, PCR primers for genotyping; **b** Genotyping of Rax::EGFP knock-in. (primer pair: Rax-FP/EGFP-RP); **c** Genotyping of Rax::EGFP knock-in. (primer pair: Rax-FP/Rax-RP); **d** Sequencing of wild type allele. (PCR product from Rax-FP/Rax-RP); **e** Sequencing of Rax::EGFP knock-in allele. (PCR product from Rax-FP/EGFP-RP). (TIFF 4455 kb) Additional file 3: Movie S1. Neuroectoderm-like structure formation from an SFEBq-cultured rESC aggregate. (MOV 17401 kb) Additional file 4: Figure S2.
**a** A representative result of RT-PCR analyses for marker expression during the differentiation process (rESC-RPC1); **b** and **c** Whole retina immunostaining for photoreceptor marker Rhodopsin (red) and grafted cell marker EGFP (green) 4w(B) and 6w(C) after subretinal transplantation in RCS rats. *Arrows* indicate the colocalization of EGFP and Rhodopsin; **d**-**g** Grafted EGFP-rESC-RPC2 integrate with the host retina 2w and 6w after transplantation. Colocalization of presynaptic markers Bassoon and Synaptophysin with EGFP expressed by donor cells in OPL was confirmed with immunostaining. *GCL* ganglion cell layer, *INL* inner nuclear layer, *ONL* outer nuclear layer. Scale bars: 50 μm; ***d'***-***g'*** The magnified images of the rectangles in **d**-**g**. *Arrow heads* indicate the colocalization of EGFP and Bassoon or Synaptophysin. *GCL* ganglion cell layer, *INL* inner nuclear layer, *OPL* outer plexiform layer, *ONL* outer nuclear layer; **h**. The ONL thickness of the retina from treated and untreated eyes. W/O: untreated retina. Data are shown as mean ± SEM, ANOVA, **P* < 0.05 and ***P* < 0.01, n = 6 for each group. (TIFF 10574 kb) Additional file 5: Table S2. List of log2 expression levels of tissue specific genes by microarray analysis. (XLS 59 kb)

## Abbreviations

AMD: Age-related macular degeneration
EB: Embryoid body
EGFP: Enhanced green fluorescent protein
ERG: Electroretinogram
ESC: Ebryonic stem cell
FBS: Fetal bovine serum
FCM: Flow cytometry
GCL: Ganglion cell layer
GO: Gene ontology
INL: Inner nuclear layer
iPS cell: Induced pluripotent stem (iPS) cell
KSR: KnockOut™ Serum Replacement
MEF: Mouse embryonic fibroblasts
ONL: Outer nuclear layer
OPL: Outer plexiform layer
PDL: poly-D-lysine
P-RPC: Primary retinal progenitor cell
RCS: Royal College of Surgeons
rESC: Rat embryonic stem cell
RP: Retinitis pigmentosa
RPC: Retinal progenitor cell
RPE: Retinal pigment epithelium
SFEBq: Quickly-aggregated/serum-free embryonic body
TALEN: Transcription activator-like effector nuclease
VEGF: Vascular endothelial growth factor

## References

[1] Klein R (2007). Overview of progress in the epidemiology of age-related macular degeneration. Ophthalmic Epidemiol.

[2] Lund RD, Ono SJ, Keegan DJ, Lawrence JM (2003). Retinal transplantation: progress and problems in clinical application. J Leukoc Biol.

[3] Pearson RA, Hippert C, Graca AB, Barber AC (2014). Photoreceptor replacement therapy: challenges presented by the diseased recipient retinal environment. Vis Neurosci.

[4] Peden MC, Suner IJ, Hammer ME, Sanderson GW (2015). Long-term outcomes in eyes receiving fixed-interval dosing of anti-vascular endothelial growth factor agents for wet age-related macular degeneration. Ophthalmology.

[5] Kucukerdonmez C, Gelisken F, Yoeruek E, Bartz-Schmidt KU, Leitritz MA (2015). Switching intravitreal anti-VEGF treatment in neovascular age-related macular degeneration. Eur J Ophthalmol.

[6] West EL, Pearson RA, MacLaren RE, Sowden JC, Ali RR (2009). Cell transplantation strategies for retinal repair. Prog Brain Res.

[7] Schwartz SD, Regillo CD, Lam BL, Eliott D, Rosenfeld PJ, Gregori NZ (2015). Human embryonic stem cell-derived retinal pigment epithelium in patients with age-related macular degeneration and Stargardt’s macular dystrophy: follow-up of two open-label phase 1/2 studies. Lancet.

[8] Lamba D, Karl M, Reh T (2008). Neural regeneration and cell replacement: a view from the eye. Cell Stem Cell.

[9] Lund RD, Wang S, Klimanskaya I, Holmes T, Ramos-Kelsey R, Lu B (2006). Human embryonic stem cell-derived cells rescue visual function in dystrophic RCS rats. Cloning Stem Cells.

[10] Lin TC, Hsu CC, Chien KH, Hung KH, Peng CH, Chen SJ (2014). Retinal stem cells and potential cell transplantation treatments. J Chin Med Assoc.

[11] Tomita M, Adachi Y, Yamada H, Takahashi K, Kiuchi K, Oyaizu H (2002). Bone marrow-derived stem cells can differentiate into retinal cells in injured rat retina. Stem cells.

[12] Decembrini S, Cananzi M, Gualdoni S, Battersby A, Allen N, Pearson RA (2011). Comparative analysis of the retinal potential of embryonic stem cells and amniotic fluid-derived stem cells. Stem Cells Dev.

[13] Gualdoni S, Baron M, Lakowski J, Decembrini S, Smith AJ, Pearson RA (2010). Adult ciliary epithelial cells, previously identified as retinal stem cells with potential for retinal repair, fail to differentiate into new rod photoreceptors. Stem Cells.

[14] MacLaren RE, Pearson RA, MacNeil A, Douglas RH, Salt TE, Akimoto M (2006). Retinal repair by transplantation of photoreceptor precursors. Nature.

[15] Cui L, Guan Y, Qu Z, Zhang J, Liao B, Ma B (2013). WNT signaling determines tumorigenicity and function of ESC-derived retinal progenitors. J Clin Invest.

[16] Decembrini S, Koch U, Radtke F, Moulin A, Arsenijevic Y (2014). Derivation of traceable and transplantable photoreceptors from mouse embryonic stem cells. Stem Cell Rep.

[17] Nakano T, Ando S, Takata N, Kawada M, Muguruma K, Sekiguchi K (2012). Self-formation of optic cups and storable stratified neural retina from human ESCs. Cell Stem Cell.

[18] Eiraku M, Takata N, Ishibashi H, Kawada M, Sakakura E, Okuda S (2011). Self-organizing optic-cup morphogenesis in three-dimensional culture. Nature.

[19] Osakada F, Ikeda H, Sasai Y, Takahashi M (2009). Stepwise differentiation of pluripotent stem cells into retinal cells. Nat Protoc.

[20] Meyer JS, Shearer RL, Capowski EE, Wright LS, Wallace KA, McMillan EL (2009). Modeling early retinal development with human embryonic and induced pluripotent stem cells. Proc Natl Acad Sci U S A.

[21] Meyer JS, Howden SE, Wallace KA, Verhoeven AD, Wright LS, Capowski EE (2011). Optic vesicle-like structures derived from human pluripotent stem cells facilitate a customized approach to retinal disease treatment. Stem Cells.

[22] Lamba DA, Gust J, Reh TA (2009). Transplantation of human embryonic stem cell-derived photoreceptors restores some visual function in Crx-deficient mice. Cell Stem Cell.

[23] Gonzalez-Cordero A, West EL, Pearson RA, Duran Y, Carvalho LS, Chu CJ (2013). Photoreceptor precursors derived from three-dimensional embryonic stem cell cultures integrate and mature within adult degenerate retina. Nat Biotechnol.

[24] Reardon S, Cyranoski D (2014). Japan stem-cell trial stirs envy. Nature.

[25] Tezel TH, Del Priore LV, Berger AS, Kaplan HJ (2007). Adult retinal pigment epithelial transplantation in exudative age-related macular degeneration. Am J Ophthalmol.

[26] Barber AC, Hippert C, Duran Y, West EL, Bainbridge JWB, Warre-Cornish K (2013). Repair of the degenerate retina by photoreceptor transplantation. Proc Natl Acad Sci U S A.

[27] Schwartz SD, Hubschman JP, Heilwell G, Franco-Cardenas V, Pan CK, Ostrick RM (2012). Embryonic stem cell trials for macular degeneration: a preliminary report. Lancet.

[28] Radtke ND, Seiler MJ, Aramant RB, Petry HM, Pidwell DJ (2002). Transplantation of intact sheets of fetal neural retina with its retinal pigment epithelium in retinitis pigmentosa patients. Am J Ophthalmol.

[29] West EL, Gonzalez-Cordero A, Hippert C, Osakada F, Martinez-Barbera JP, Pearson RA (2012). Defining the integration capacity of embryonic stem cell-derived photoreceptor precursors. Stem Cells.

[30] Arnhold S, Klein H, Semkova I, Addicks K, Schraermeyer U (2004). Neurally selected embryonic stem cells induce tumor formation after long-term survival following engraftment into the subretinal space. Invest Ophthalmol Vis Sci.

[31] Bennett J (2007). Retinal progenitor cells - timing is everything. New Engl J Med.

[32] Aitman TJ, Critser JK, Cuppen E, Dominiczak A, Fernandez-Suarez XM, Flint J (2008). Progress and prospects in rat genetics: a community view. Nat Genet.

[33] Jacob HJ, Kwitek AE (2002). Rat genetics: Attaching physiology and pharmacology to the genome. Nat Rev Genet.

[34] Evans MJ, Kaufman MH (1981). Establishment in culture of pluripotential cells from mouse embryos. Nature.

[35] Thomson JA, Itskovitz-Eldor J, Shapiro SS, Waknitz MA, Swiergiel JJ, Marshall VS (1998). Embryonic stem cell lines derived from human blastocysts. Science.

[36] Buehr M, Meek S, Blair K, Yang J, Ure J, Silva J (2008). Capture of authentic embryonic stem cells from rat blastocysts. Cell.

[37] Li P, Tong C, Mehrian-Shai R, Jia L, Wu N, Yan Y (2008). Germline competent embryonic stem cells derived from rat blastocysts. Cell.

[38] Cao N, Liao J, Liu ZM, Zhu WM, Wang J, Liu LJ (2011). In vitro differentiation of rat embryonic stem cells into functional cardiomyocytes. Cell Res.

[39] Zhao XY, Lv Z, Liu L, Wang L, Tong M, Zhou Q (2010). Derivation of embryonic stem cells from Brown Norway rats blastocysts. J Genet Genomics.

[40] Wang Z, Sheng C, Li T, Teng F, Sang L, Cao F (2012). Generation of tripotent neural progenitor cells from rat embryonic stem cells. J Genet Genomics.

[41] Tong C, Li P, Wu NL, Yan YZ, Ying QL (2010). Production of p53 gene knockout rats by homologous recombination in embryonic stem cells. Nature.

[42] Zhang J, Li H, Wu Z, Tan XJ, Liu FY, Huang XH (2013). Differentiation of rat iPS cells and ES cells into granulosa cell-like cells in vitro. Acta Biochim Biophys Sin (Shanghai).

[43] Nichols J, Smith A, Buehr M (1998). Rat and mouse epiblasts differ in their capacity to generate extraembryonic endoderm. Reprod Fert Develop.

[44] Brederlau A, Correia AS, Anisimov SV, Elmi M, Paul G, Roybon L (2006). Transplantation of human embryonic stem cell-derived cells to a rat model of Parkinson’s disease: effect of in vitro differentiation on graft survival and teratoma formation. Stem Cells.

[45] Coffey PJ, Girman S, Wang SM, Hetherington L, Keegan DJ, Adamson P (2002). Long-term preservation of cortically dependent visual function in RCS rats by transplantation. Nat Neurosci.

[46] Doggrell SA, Brown L (1998). Rat models of hypertension, cardiac hypertrophy and failure. Cardiovasc Res.

[47] Zhang JF, Wu YL, Jin Y, Ji F, Sinclair SH, Luo Y (2008). Intravitreal injection of erythropoietin protects both retinal vascular and neuronal cells in early diabetes. Invest Ophthalmol Vis Sci.

[48] Bourne MC, Campbell DA, Tansley K (1938). Hereditary degeneration of the rat retina. Br J Ophthalmol.

[49] Dowling JE, Sidman RL (1962). Inherited retinal dystrophy in the rat. J Cell Biol.

[50] LaVail MM (1981). Photoreceptor characteristics in congenic strains of RCS rats. Invest Ophthalmol Vis Sci.

[51] D’Cruz PM, Yasumura D, Weir J, Matthes MT, Abderrahim H, LaVail MM (2000). Mutation of the receptor tyrosine kinase gene Mertk in the retinal dystrophic RCS rat. Hum Mol Genet.

[52] Gal A, Li Y, Thompson DA, Weir J, Orth U, Jacobson SG (2000). Mutations in MERTK, the human orthologue of the RCS rat retinal dystrophy gene, cause retinitis pigmentosa. Nat Genet.

[53] Rivas MA, Vecino E (2009). Animal models and different therapies for treatment of retinitis pigmentosa. Histol Histopathol.

[54] Lu B, Malcuit C, Wang S, Girman S, Francis P, Lemieux L (2009). Long-term safety and function of RPE from human embryonic stem cells in preclinical models of macular degeneration. Stem Cells.

[55] Hockemeyer D, Wang HY, Kiani S, Lai CS, Gao Q, Cassady JP (2011). Genetic engineering of human pluripotent cells using TALE nucleases. Nat Biotechnol.

[56] Sauve Y, Pinilla I, Lund RD (2006). Partial preservation of rod and cone ERG function following subretinal injection of ARPE-19 cells in RCS rats. Vis Res.

[57] Huang DW, Sherman BT, Lempicki RA (2009). Systematic and integrative analysis of large gene lists using DAVID bioinformatics resources. Nat Protoc.

[58] Hruska K, Mathew S, Lund R, Fang Y, Sugatani T (2011). Cardiovascular risk factors in chronic kidney disease: does phosphate qualify?. Kidney Int Suppl.

[59] Eiraku M, Sasai Y (2012). Mouse embryonic stem cell culture for generation of three-dimensional retinal and cortical tissues. Nat Protoc.

[60] Eiraku M, Watanabe K, Matsuo-Takasaki M, Kawada M, Yonemura S, Matsumura M (2008). Self-organized formation of polarized cortical tissues from ESCs and its active manipulation by extrinsic signals. Cell Stem Cell.

[61] Chen B, Dodge ME, Tang W, Lu J, Ma Z, Fan CW (2009). Small molecule-mediated disruption of Wnt-dependent signaling in tissue regeneration and cancer. Nat Chem Biol.

[62] Zuber ME, Gestri G, Viczian AS, Barsacchi G, Harris WA (2003). Specification of the vertebrate eye by a network of eye field transcription factors. Development.

[63] Lakowski J, Han YT, Pearson RA, Gonzalez-Cordero A, West EL, Gualdoni S (2011). Effective transplantation of photoreceptor precursor cells selected via cell surface antigen expression. Stem Cells.

[64] Lamba DA, Reh TA (2011). Microarray characterization of human embryonic stem cell--derived retinal cultures. Invest Ophthalmol Vis Sci.

[65] Jacob HJ (1999). Functional genomics and rat models. Genome Res.

[66] Carr AJ, Vugler AA, Hikita ST, Lawrence JM, Gias C, Chen LL (2009). Protective effects of human iPS-derived retinal pigment epithelium cell transplantation in the retinal dystrophic rat. PLoS One.

[67] Farahpour MR, Ghayour SJ (2014). Effect of in situ delivery of acetyl-L-carnitine on peripheral nerve regeneration and functional recovery in transected sciatic nerve in rat. Int J Surg.

[68] Bohm MR, Prokosch V, Bruckner M, Pfrommer S, Melkonyan H, Thanos S (2015). betaB2-crystallin promotes axonal regeneration in the injured optic nerve in adult rats. Cell Transplant.

[69] Zhang J, Hu LM, Xu G, Wu Y, Shen J, Luo Y (2010). Anti-VEGF effects of intravitreal erythropoietin in early diabetic retinopathy. Front Biosci.

[70] Gu L, Xu H, Wang F, Xu G, Sinha D, Wang J (2014). Erythropoietin exerts a neuroprotective function against glutamate neurotoxicity in experimental diabetic retina. Invest Ophthalmol Vis Sci.

[71] Guan Y, Cui L, Qu Z, Lu L, Wang F, Wu Y (2013). Subretinal transplantation of rat MSCs and erythropoietin gene modified rat MSCs for protecting and rescuing degenerative retina in rats. Curr Mol Med.

[72] Atanur SS, Diaz AG, Maratou K, Sarkis A, Rotival M, Game L (2013). Genome sequencing reveals loci under artificial selection that underlie disease phenotypes in the laboratory rat. Cell.

[73] Lamba DA, Karl MO, Ware CB, Reh TA (2006). Efficient generation of retinal progenitor cells from human embryonic stem cells. Proc Natl Acad Sci U S A.

[74] Turner DL, Cepko CL (1987). A common progenitor for neurons and glia persists in rat retina late in development. Nature.

[75] Centanin L, Hoeckendorf B, Wittbrodt J (2011). Fate restriction and multipotency in retinal stem cells. Cell Stem Cell.

[76] Wetts R, Fraser SE (1988). Multipotent precursors can give rise to all major cell types of the frog retina. Science.

[77] Cepko C (2014). Intrinsically different retinal progenitor cells produce specific types of progeny. Nat Rev Neurosci.

[78] Hu Y, Ji J, Xia J, Zhao P, Fan X, Wang Z (2013). An in vitro comparison study: the effects of fetal bovine serum concentration on retinal progenitor cell multipotentiality. Neurosci Lett.

[79] Santos-Ferreira T, Postel K, Stutzki H, Kurth T, Zeck G, Ader M (2015). Daylight vision repair by cell transplantation. Stem Cells.

[80] Luo J, Baranov P, Patel S, Ouyang H, Quach J, Wu F (2014). Human retinal progenitor cell transplantation preserves vision. J Biol Chem.

[81] Peachey NS, Ball SL (2003). Electrophysiological analysis of visual function in mutant mice. Doc Ophthalmol.

[82] Pinilla I, Lund RD, Sauve Y (2004). Contribution of rod and cone pathways to the dark-adapted electroretinogram (ERG) b-wave following retinal degeneration in RCS rats. Vis Res.

[83] Pinilla I, Lund RD, Lu B, Sauve Y (2005). Measuring the cone contribution to the ERG b-wave to assess function and predict anatomical rescue in RCS rats. Vis Res.

[84] Gamm DM, Wang S, Lu B, Girman S, Holmes T, Bischoff N (2007). Protection of visual functions by human neural progenitors in a rat model of retinal disease. PLoS One.

[85] Lund RD, Wang S, Lu B, Girman S, Holmes T, Sauve Y (2007). Cells isolated from umbilical cord tissue rescue photoreceptors and visual functions in a rodent model of retinal disease. Stem Cells.

[86] Tian N, Slaughter MM (1995). Correlation of dynamic responses in the ON bipolar neuron and the b-wave of the electroretinogram. Vis Res.

[87] Perlman I. The Electroretinogram: ERG. 2001 May 1 [Updated 2007 Jun 27]. In: Kolb H, Fernandez E, Nelson R, editors. Webvision: The Organization of the Retina and Visual System [Internet]. Salt Lake City (UT): University of Utah Health Sciences Center; 1995. Available from: http://www.ncbi.nlm.nih.gov/books/NBK11554/. 21413407

[88] Cuenca N, Pinilla I, Sauve Y, Lund R (2005). Early changes in synaptic connectivity following progressive photoreceptor degeneration in RCS rats. Eur J Neurosci.

[89] Jian Q, Xu H, Xie H, Tian C, Zhao T, Yin Z (2009). Activation of retinal stem cells in the proliferating marginal region of RCS rats during development of retinitis pigmentosa. Neurosci Lett.

[90] Yamazaki H, Ohguro H, Maeda T, Maruyama I, Takano Y, Metoki T (2002). Preservation of retinal morphology and functions in royal college surgeons rat by nilvadipine, a Ca(2+) antagonist. Invest Ophthalmol Vis Sci.

[91] Vollrath D, Feng W, Duncan JL, Yasumura D, D’Cruz PM, Chappelow A (2001). Correction of the retinal dystrophy phenotype of the RCS rat by viral gene transfer of Mertk. Proc Natl Acad Sci U S A.

[92] Tschernutter M, Schlichtenbrede FC, Howe S, Balaggan KS, Munro PM, Bainbridge JW (2005). Long-term preservation of retinal function in the RCS rat model of retinitis pigmentosa following lentivirus-mediated gene therapy. Gene Ther.

[93] Jian Q, Li Y, Yin ZQ (2015). Rat BMSCs initiate retinal endogenous repair through NGF/TrkA signaling. Exp Eye Res.

[94] Machida S, Chaudhry P, Shinohara T, Singh DP, Reddy VN, Chylack LT (2001). Lens epithelium-derived growth factor promotes photoreceptor survival in light-damaged and RCS rats. Invest Ophthalmol Vis Sci.

[95] Sheedlo HJ, Li L, Turner JE (1991). Photoreceptor cell rescue at early and late RPE-cell transplantation periods during retinal disease in RCS dystrophic rats. J Neural Transplant Plast.

[96] Sauve Y, Girman SV, Wang S, Keegan DJ, Lund RD (2002). Preservation of visual responsiveness in the superior colliculus of RCS rats after retinal pigment epithelium cell transplantation. Neuroscience.

[97] Wang S, Girman S, Lu B, Bischoff N, Holmes T, Shearer R (2008). Long-term vision rescue by human neural progenitors in a rat model of photoreceptor degeneration. Invest Ophthalmol Vis Sci.

[98] McGill TJ, Cottam B, Lu B, Wang S, Girman S, Tian C (2012). Transplantation of human central nervous system stem cells - neuroprotection in retinal degeneration. Eur J Neurosci.

[99] Cuenca N, Fernandez-Sanchez L, McGill TJ, Lu B, Wang S, Lund R (2013). Phagocytosis of photoreceptor outer segments by transplanted human neural stem cells as a neuroprotective mechanism in retinal degeneration. Invest Ophthalmol Vis Sci.

