# Supplementary material for: Transplantation of rat embryonic stem cell-derived retinal progenitor cells preserves the retinal structure and function in rat retinal degeneration
Source: Stem Cell Res Ther. 2015 Nov 9;6:219. doi: 10.1186/s13287-015-0207-x (PMC4640237; doi:10.1186/s13287-015-0207-x)
Supplement: Additional file 1: Table S1. — PCR primer sequences. (DOC 53 kb) [file 13287_2015_207_MOESM1_ESM.doc]

**Additional file**

**Table S1.** PCR primer sequences

| Oct4 F | TGGAGGGATGGCATACTGTGGAC |
| --- | --- |
| Oct4 R | GCTCTAGCTCCTTCTGCAGGGCTT |
| Nanog F | TCAGGCCCAGTTGTGTGCACTC |
| Nanog R | TGAGCCCTTCTGAGTCAGGCCG |
| Sox2 F | ATCACAACAATCGCGGCGGC |
| Sox2 R | CACGGCCTTGCGCGGAGATC |
| Pax6 F | AGCCCAGTTTTCAGAGCCACGT |
| Pax6 R | CAGTGGCCGCCCGTTGACAA |
| Nesin F | AAGTTCCAGCTGGCTGTGGAAGCC |
| Nesin R | CGTCCAGGTGTCTGCAACCGA |
| Six3 F | TAAGAACCGGCGACAGCGCG |
| Six3 R | CGCTCCGTCAGGCTGGACAC |
| Six6 F | CCGTCCACGCCAGCGCTTTA |
| Six6 R | TCTGAGTGGGCGAGGGCCTC |
| Lhx2 F | CTACAACGCCGCGCTGAGCTG |
| Lhx2 R | TGGCCCGGGCATTCTGAAACC |
| GAPDH F | TTCTTGTGCAGTGCCAGCCTCGT |
| GAPDH R | TGGGTTTCCCGTTGATGACCAGC |
| Crx F | GTCCCATACTCAAGTGCCCCTAGGA |
| Crx R | TCGCCCTGCGATTCTTGAACCA |
| Rax F | TTCGAAGCTACTCGCCCCTGC |
| Rax R | GGCCAACTCTTCGCGGCTGT |
| Vsx2 F | CTGGCCCCCGGGCATTTGTT |
| Vsx2 R | TGCAAGTGGACGCTCTGCGG |
| Gfap F | GCGGGAGTCGGCGAGTTACC |
| Gfap R | CCAGGCTGGTTTCTCGGATCTGGA |
| Map2 F | GCTCAGGCTCCCAGTGCGTT |
| Map2 R | TCTGACCTGGTGGTCCGTCG |
| Nrl F | GGCCGATGAGGTTCTGGGGC |
| Nrl R | ACTCGCGCACCGACATCGAG |
| Otx2-F | TTTTCAAGCGTCCAATGCGG |
| Otx2-R | TTGGAGTGACGGAACTCTGG |
| Ascl1-F | CCACCATCTCCCCCAACTAC |
| Ascl1-R | CCTGCCATCCTGCTTCCAAAG |
| Rcvrn-F | TACGACGTAGACGGCAATGG |
| Rcvrn-R | TTCCTCCTCTGTAAGTTTATCATCA |
| Rho-F | ACCCTTGGAGGTGAAATCGG |
| Rho-R | CACATGAACACTGCATGCCC |
| Prox1-F | GGAAGCGCAATGAAGGGCTAT |
| Prox1-F | GGAAGCGCAATGAAGGGCTAT |
| Prox1-R | AGTTCCTCTGTGCTGGTGAC |
| Prkca-F | CCATGGCTGACGTTTACCCG |
| Prkca-R | AGATGAAGTCGGTGCAGTGG |
| Atoh7-F | AGCTGTCCAAGTACGAGACAC |
| Atoh7-R | TCTGGCTGGAAACCGAAGAA |
| Tuj1 F | CGTCTCTAGCCGAGTGAAGT |
| Tuj1 R | GGGCACATACTTGTGAGAGGA |
