# Supplementary figures and images for: Transplantation of rat embryonic stem cell-derived retinal progenitor cells preserves the retinal structure and function in rat retinal degeneration
Source: Stem Cell Res Ther. 2015 Nov 9;6:219. doi: 10.1186/s13287-015-0207-x (PMC4640237; doi:10.1186/s13287-015-0207-x)

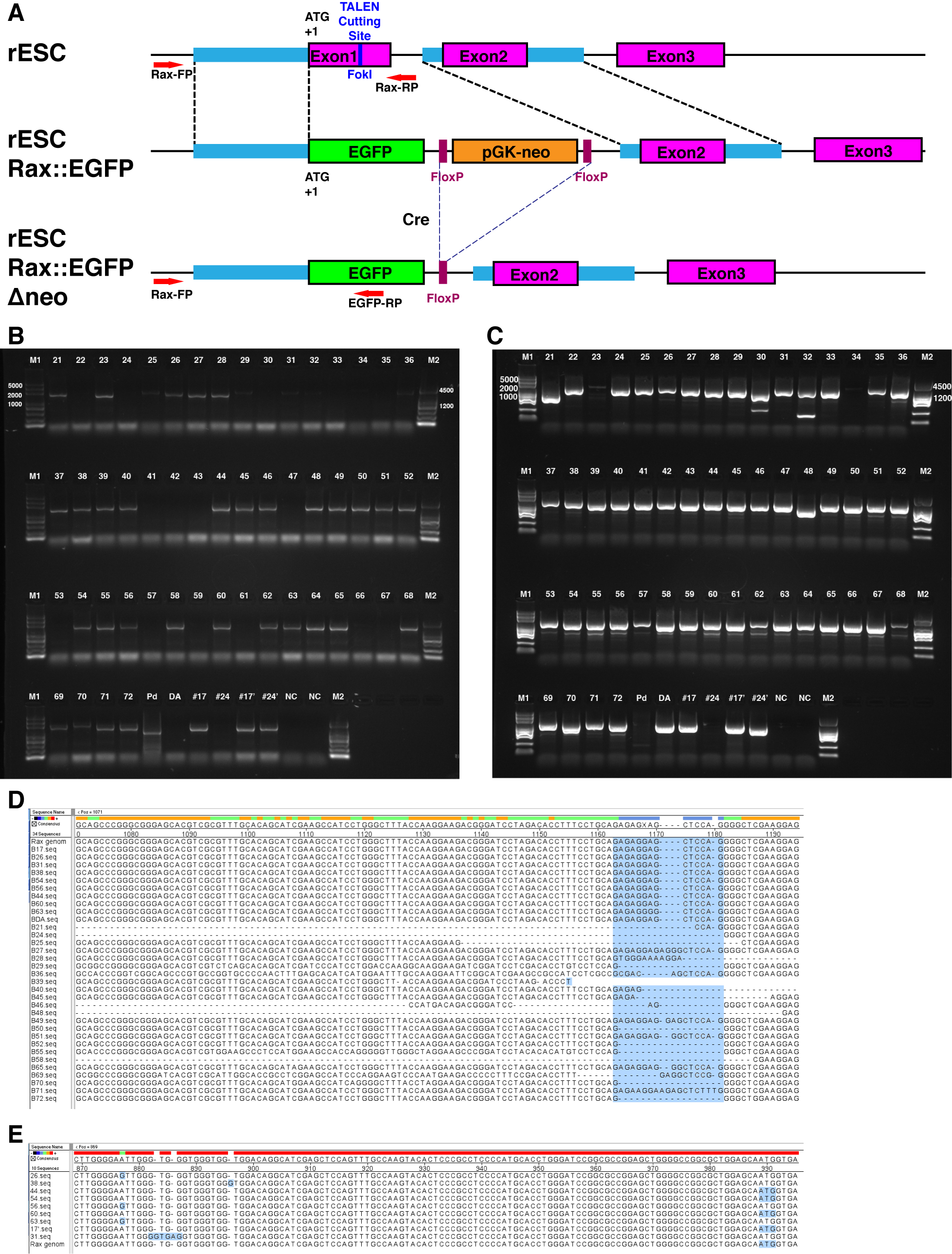

Supplement: Additional file 2: Figure S1. — Construction of Rax::EGFP reporter rESCs. a Knock-in strategy for the Rax::EGFP reporter construct. Red arrows, PCR primers for genotyping; b Genotyping of Rax::EGFP knock-in. (primer pair: Rax-FP/EGFP-RP); c Genotyping of Rax::EGFP knock-in. (primer pair: Rax-FP/Rax-RP); d Sequencing of wild type allele. (PCR product from Rax-FP/Rax-RP); e Sequencing of Rax::EGFP knock-in allele. (PCR product from Rax-FP/EGFP-RP). (TIFF 4455 kb) [file 13287_2015_207_MOESM2_ESM.tiff]

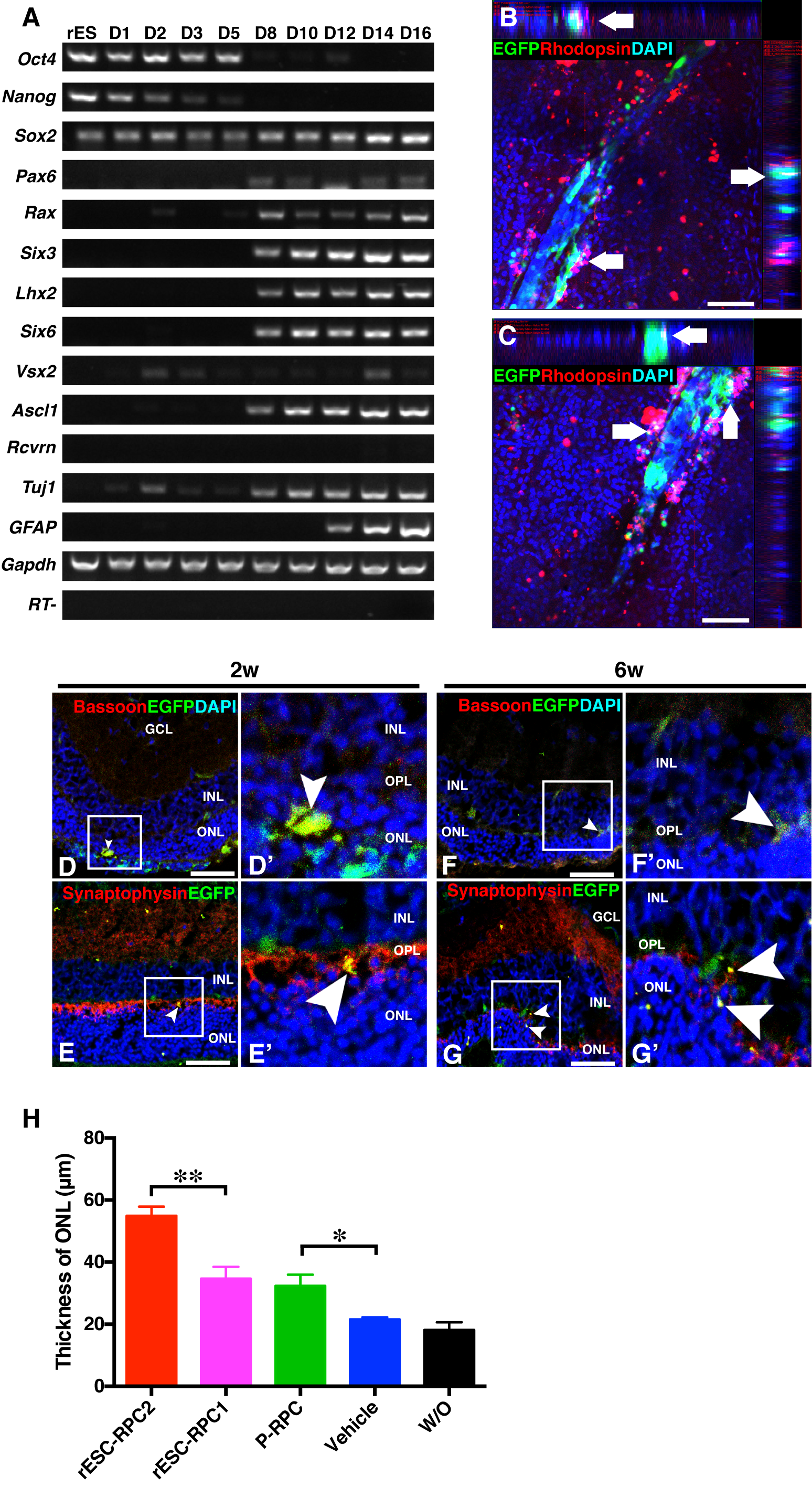

Supplement: Additional file 4: Figure S2. — a A representative result of RT-PCR analyses for marker expression during the differentiation process (rESC-RPC1); b and c Whole retina immunostaining for photoreceptor marker Rhodopsin (red) and grafted cell marker EGFP (green) 4w(B) and 6w(C) after subretinal transplantation in RCS rats. Arrows indicate the colocalization of EGFP and Rhodopsin; d-g Grafted EGFP-rESC-RPC2 integrate with the host retina 2w and 6w after transplantation. Colocalization of presynaptic markers Bassoon and Synaptophysin with EGFP expressed by donor cells in OPL was confirmed with immunostaining. GCL ganglion cell layer, INL inner nuclear layer, ONL outer nuclear layer. Scale bars: 50 μm; d'-g' The magnified images of the rectangles in d-g. Arrow heads indicate the colocalization of EGFP and Bassoon or Synaptophysin. GCL ganglion cell layer, INL inner nuclear layer, OPL outer plexiform layer, ONL outer nuclear layer; h. The ONL thickness of the retina from treated and untreated eyes. W/O: untreated retina. Data are shown as mean ± SEM, ANOVA, *P < 0.05 and **P < 0.01, n = 6 for each group. (TIFF 10574 kb) [file 13287_2015_207_MOESM4_ESM.tiff]
